# Allergen induces pulmonary neuroendocrine cell hyperplasia in a model of asthma

**DOI:** 10.1172/jci.insight.187018

**Published:** 2025-07-08

**Authors:** Estelle Kim, Brian K. Wells, Hannah Indralingam, Yujuan Su, Jamie Verheyden, Xin Sun

**Affiliations:** 1 Department of Biological Sciences and; 2 Department of Pediatrics, University of California, San Diego (UCSD), La Jolla, California, USA.

**Keywords:** Cell biology, Development, Pulmonology, Asthma, Mouse models, Neuroendocrine regulation

## Abstract

Asthma is characterized by exacerbated response to triggers such as allergen. While pulmonary neuroendocrine cells (PNECs), a rare population of airway epithelial cells, are essential for amplifying allergen-induced asthma response, how PNECs are regulated to achieve this role remains poorly understood. Here we show that in the adult mouse airway, inactivation of achaete-scute-like protein 1 gene in PNECs led to loss of these cells. Intriguingly, exposure of these mutants to house dust mites (HDM), a common allergen, led to reappearance of PNECs. Similarly, exposure of wild-type mice to HDM led to PNEC hyperplasia, a result of proliferation of existing PNECs and transdifferentiation from club cells. Single-cell RNA-Seq experiments revealed PNEC heterogeneity, including the emergence of an allergen-induced PNEC subtype. Notch signaling was downregulated in HDM-treated airway, and treatment with Notch agonist prevented PNEC hyperplasia. These findings together suggest that HDM-induced PNEC hyperplasia may contribute to exacerbated asthma response.

## Abstract 2

Asthma is triggered by allergen exposure. Aside from known responses, allergen triggers transdifferentiation of club cells into pulmonary neuroendocrine cells, which contribute to disease symptoms.

## Introduction

Environmental insults such as allergen are a primary trigger for asthma, a prevalent airway disorder. Repeated lung exposure to allergen results in increased immune infiltrates, goblet cell metaplasia, and exacerbated bronchoconstriction, hallmarks of asthma. An unexpected cell type that plays a central role in lung response to allergen is pulmonary neuroendocrine cells (PNECs), a rare population that represents less than 1% of airway epithelial cells (1, 2). PNECs are specialized epithelial cells that sense aerosol or mechanical signals and, in turn, secrete neuropeptides and neurotransmitters. We found that in a mouse mutant, preventing PNEC formation starting in early embryonic development led to a blunted response to allergen postnatally (3). Data from the mutant suggest that PNECs act through neuropeptides, such as calcitonin gene-related peptide (CGRP), to stimulate innate lymphoid group 2 cells (ILC2s), thereby triggering the cascade of type 2 immune response. Cytokines such as IL-13 secreted from immune cells, together with γ-aminobutyric acid, a neurotransmitter produced by PNECs, induce the conversion of club cells into mucus-producing goblet cells (3). In a more recent study, we mapped a complete neural circuit that originates from the lung, signals to brainstem neurons, and feeds back to control allergen-induced airway constriction (4). Given that PNECs are also a primary airway cell type that are innervated by both afferent and efferent nerves, these findings together suggest that they play a key role in lung function as a sensory organ (1, 4).

PNEC hyperplasia has been documented in many lung diseases, including allergic asthma (1, 3, 5). However, the cellular and molecular mechanisms leading to such pathological change have not been elucidated. Achaete-scute-like protein 1 (*Ascl1*), a basic helix-loop-helix transcription factor, is an early marker of PNEC fate. Inactivation of *Ascl1* in mice prevented the formation of these cells (6, 7), demonstrating that *Ascl1* is essential for PNEC development. However, its role during homeostasis and asthma pathogenesis has not been addressed.

*Ascl1* expression is negatively regulated by Notch signaling (8–10). In mouse development, early inhibition of Notch signaling led to *Ascl1* expression and the differentiation of primordial airway precursors toward PNEC fate (6, 11, 12). This was corroborated in induced pluripotent stem cell–derived airway progenitor culture, where reduced Notch signaling allowed *Ascl1* expression, which in turn induced PNEC formation (13, 14). While Notch function has been studied during development, whether it plays a role in allergen-induced PNEC hyperplasia has not been addressed.

In this study, we show that inactivation of *Ascl1*, specifically in PNECs, led to rapid loss of these cells due to cell death. Unexpectedly, PNECs reappeared in these mutants following allergen challenge. Similar PNEC hyperplasia was found in wild-type (WT) mice exposed to allergen, a result of both PNEC proliferation and club cell transdifferentiation. Single-cell RNA-Seq (scRNA-Seq) revealed heterogeneity among PNECs, including solitary PNECs versus those clustered together, forming neuroepithelial bodies (NEBs). Allergen exposure accentuated such heterogeneity by inducing a new subtype. We found that allergen exposure led to downregulation of Notch signaling in the proximal airway. Administration of a Notch agonist reversed PNEC hyperplasia, suggesting that modulating Notch signaling may prevent PNEC hyperplasia in the asthmatic airway.

## Results

### Ascl1 is required for PNEC maintenance during homeostasis.

**Figure 1:**
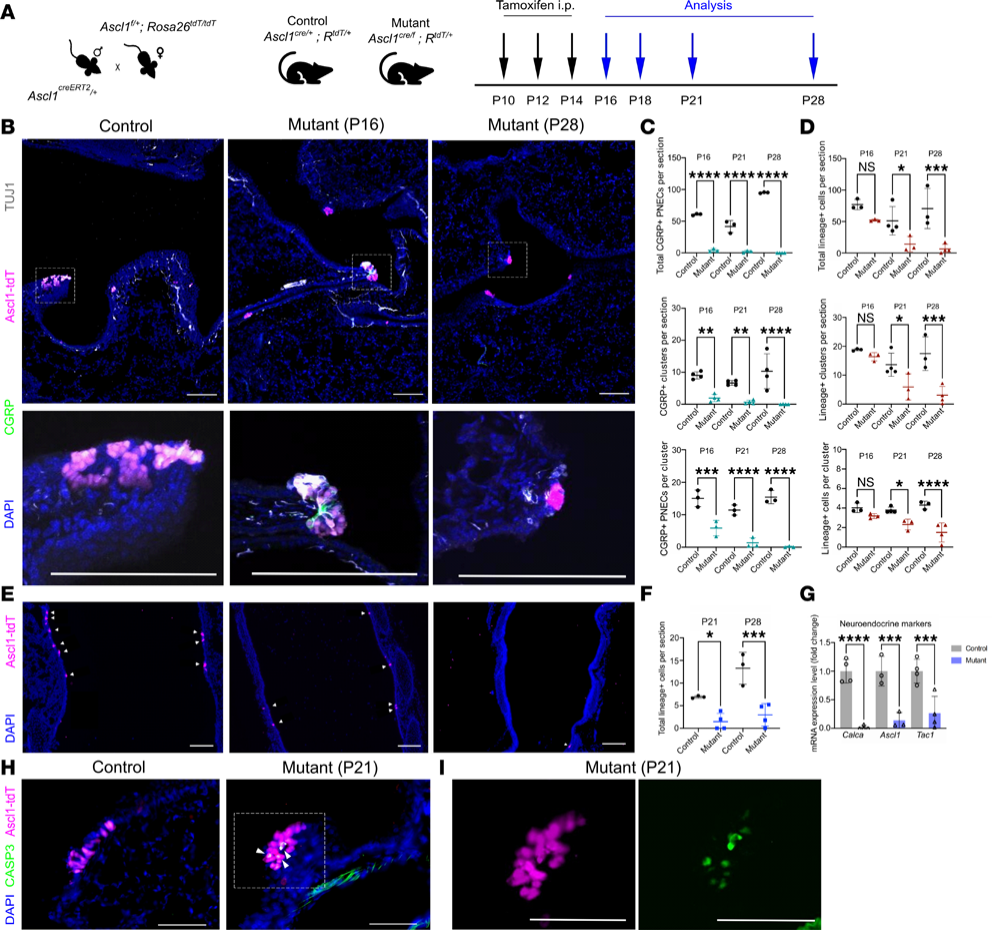
*Ascl1* is required for PNEC maintenance. (**A**) Schematic of tamoxifen administration to generate *Ascl1*-mutant mice. (**B**) Representative images of control (*Ascl1^creERT2/+^ Rosa26^tdTomato/+^*) and mutant (*Ascl1^cre/fl^ Rosa26^tdTomato/+^*) lung sections stained with anti-CGRP and anti-TUJ1 antibodies to characterize neuropeptide expression and innervation of tdTomato-lineaged PNECs, respectively. (**C** and **D**) Quantification of PNEC numbers based on CGRP staining or *Ascl1* lineage at indicated ages. For this and all cell number quantifications that follow, each dot represents the average value across multiple sections representing the full range of depths from 1 lung (20–25 sections per lung, 10–15 sections per trachea), unless otherwise indicated. (**E**) Representative images of control and mutant trachea sections of *Ascl1*-lineaged cells. (**F**) Quantification of *Ascl1*-lineaged cells in the trachea. (**G**) Relative transcript level of neuroendocrine markers as assayed by qRT-PCR in P16 whole lungs. (**H**) Representative images of lung sections stained with anti–cleaved caspase-3 to label apoptotic cells (white arrowheads) in *Ascl1*-lineaged cells. (**I**) Close-up of boxed area from mutant lung (P21) from **H**. One-way ANOVA was used for **C** and **D** (*n* = 3–4 for each group). Student’s *t* test was used for **F** (*n* = 3 for each group). * for *P* < 0.05, ** for *P* < 0.01, *** for *P* < 0.001, and **** for *P* < 0.0001. Error bars represent mean ± SD. Scale bar size 100 μm for **B** and **E**; 50 μm for **H** and **I**. TUJ1, beta III tubulin; qRT, quantitative reverse transcription.

While inactivation of *Ascl1* at the start of lung development prevented PNEC formation (3), whether *Ascl1* is also required for PNEC maintenance remains unclear. To address this, we inactivated *Ascl1* in PNECs postnatally by generating *Ascl1^creERT2/fl^* mutant and *Ascl1^creERT2/+^* control mice, also carrying a cre reporter (*Rosa26^tdTomato^* or *Rosa26^ZsGreen^*), to follow *Ascl1*-lineaged cells. Tamoxifen was injected at postnatal day 10 (P10), P12, and P14 to inactivate *Ascl1* in PNECs (Figure 1A). This led to greatly reduced *Ascl1*-lineaged cells at P28, preceded by reduced expression of PNEC markers CGRP, synaptophysin (SYP), and synaptotagmin (SYT1) as early as P16 (Figure 1B and Supplemental Figures 1–3; supplemental material available online with this article; https://doi.org/10.1172/jci.insight.187018DS1). Quantification of total number of PNECs per section, number of clusters per section, and cells per cluster verified a significant loss in PNEC number, based on both antibody staining and *Ascl1*-lineage labeling (Figure 1, C and D, and Supplemental Figure 3, D and E). Loss of PNECs was also observed in the trachea (Figure 1, E–G, and Supplemental Figure 4, A and B). Whole lung qRT-PCR analysis of PNEC marker genes also verified the reduction (Figure 1G). In contrast, other airway cell type markers, including those for basal, club, ciliated, and goblet cells, were unchanged (Supplemental Figure 3C and Supplemental Figure 4C). The apoptotic marker, cleaved caspase-3, was detected in *Ascl1*-lineaged PNECs in the mutant but not in the control (Figure 1, H and I, and Supplemental Figure 1B). These results together demonstrate that *Ascl1* is required for the maintenance and survival of PNECs in the postnatal airway.

### PNECs in the Ascl1 mutant recover in number following allergen challenge.

**Figure 2:**
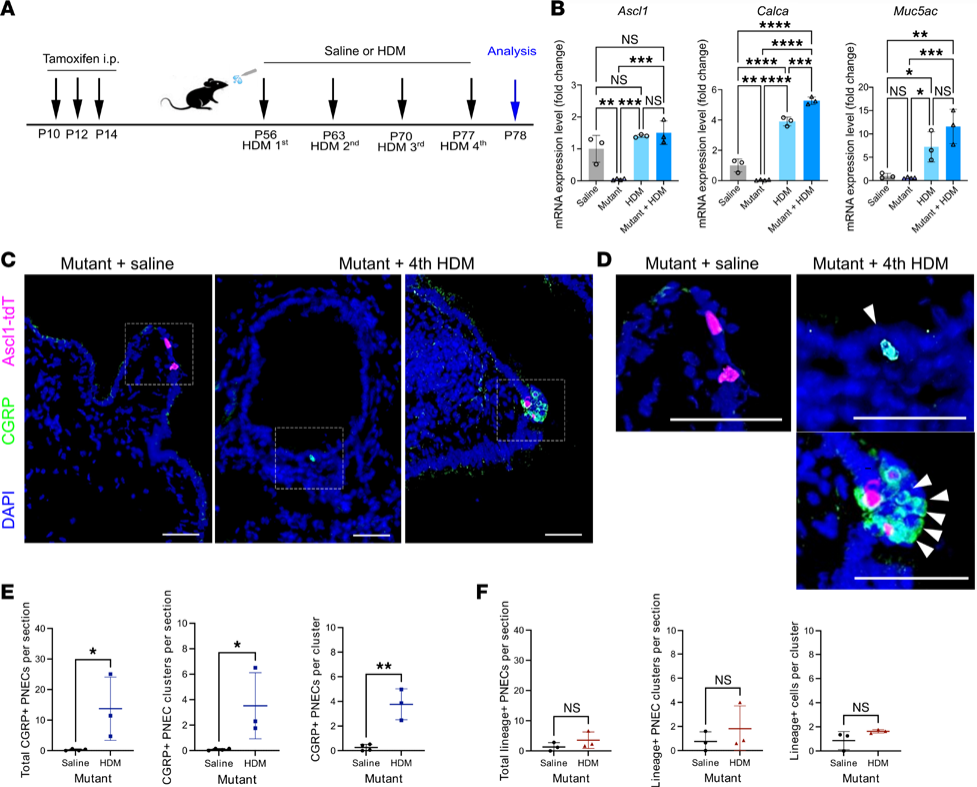
Allergen challenge induces ectopic PNECs in *Ascl1* mutants. (**A**) Schematic of HDM administration to *Ascl1*-mutant mice. (**B**) qRT-PCR of whole lungs at P78. Two-way ANOVA was used (*n* = 3 or 4 lungs for each group). (**C**) Representative images of *Ascl1*-mutant lung sections stained with anti-CGRP antibody. (**D**) Boxed areas magnified from **C** showing the emergence of nonlineaged PNECs following allergen challenge (white arrowheads). (**E** and **F**) Quantitative analyses of PNEC numbers based on CGRP staining or *Ascl1*-lineaged PNECs. Student’s *t* test was used (*n* = 3–4 lungs for each group). Each dot represents the average value of multiple sections from 1 lung. * for *P* < 0.05, ** for *P* < 0.01, *** for *P* < 0.001, and **** for *P* < 0.0001. Error bars represent mean ± SD. Scale bar size 50 μm.

We have shown previously that blocking PNEC formation during development using the pan-epithelial *Shh^cre^* led to blunted response to allergen, including dampened goblet cell metaplasia (3). This was verified again in *Shh^cre^ Ascl1^fl/fl^* mutants following house dust mite (HDM) challenge (Supplemental Figure 5). Since *Ascl1^creERT2/fl^* mutants are also devoid of PNECs, when we exposed them to HDM, we expected similar reduced response (Figure 2A). Instead, by mucin 5AC (MUC5AC) antibody staining as well as *Muc5ac* qRT-PCR, *Ascl1^creERT2/fl^* mutant mice exposed to HDM showed a robust goblet cell metaplasia response, similar to that in the control genotype (Figure 2B and Supplemental Figure 5).

To address what may underlie this result, we assayed for PNECs in the *Ascl1^creERT2/fl^ Rosa26^tdTomato/+^* mutant. In saline control, few remaining *Ascl1*-lineaged cells lacked CGRP expression (Figure 2, C and D). Interestingly, CGRP^+^ cells were readily detectable in allergen response; however, they were not *Ascl1*-lineaged (Figure 2, C–F). The newly emerged cells also expressed other PNEC markers, SYP and SYT1, supporting that they are PNECs (Supplemental Figure 6). In contrast, in the *Shh^cre^ Ascl1^fl/fl^* mutant, where *Ascl1* is inactivated in all lung epithelial cells, no PNECs reemerged following allergen challenge (Supplemental Figure 5). These results together suggest that PNECs, while depleted in *Ascl1^creERT2/fl^* lungs, were induced by allergen challenge from other epithelial progenitors. These ectopic PNECs are capable of eliciting allergen-induced goblet cell metaplasia response.

To further assess whether these newly emergent PNECs mount a functional allergic response, we performed airway hyperresponsiveness (AHR) measurements using flexiVent after 4 doses of HDM (Supplemental Figure 7A). The *Ascl1^creERT2/fl^* mutants exhibited robust AHR, even higher than control mice treated with HDM, though the differences were not statistically significant. In addition, immune profiling revealed a robust increase in recruitment of type 2 immune cells in the *Ascl1^creERT2/fl^* mutants following HDM challenge compared with saline. For some cell types (e.g., Th2 cells), the increase was similar to control with HDM, while for other cell types (e.g., eosinophils and ILC2s), the increase was higher than control with HDM (Supplemental Figure 7B). These findings suggest that, despite their distinct origin, the newly transdifferentiated PNECs in the *Ascl1^creERT2/fl^* airway can support the allergen-induced immune response to a comparable or greater extent compared with that of WT mouse lungs.

### PNECs increase in number in WT lungs following allergen challenge.

**Figure 3:**
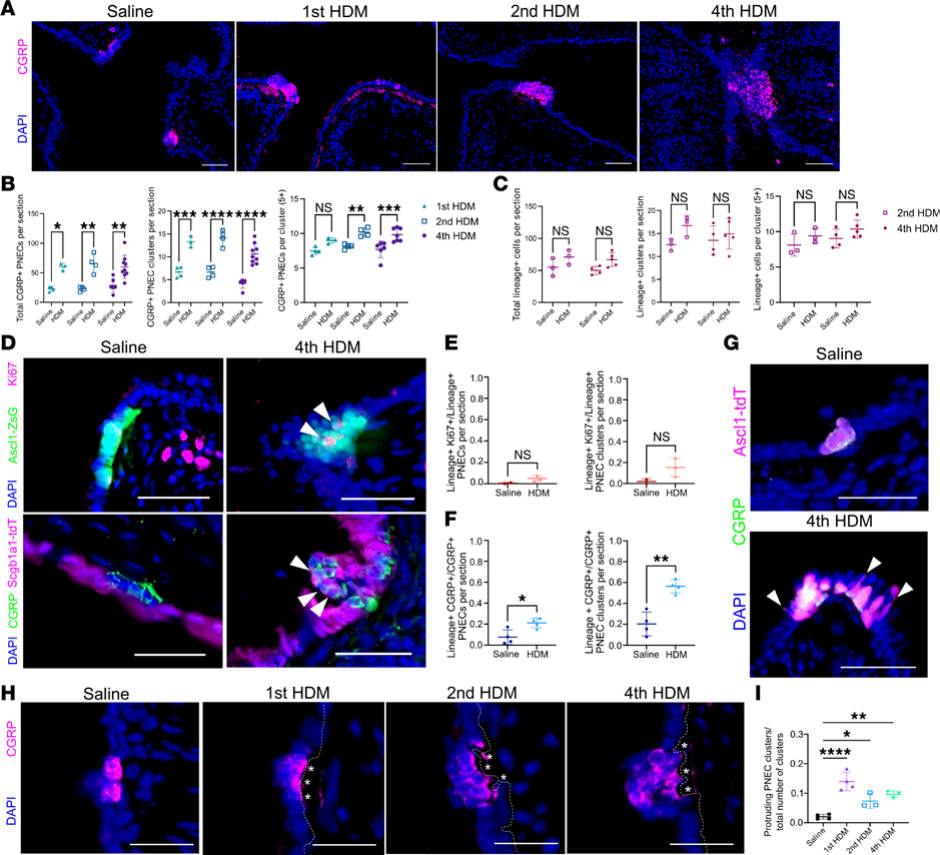
Proliferation and club cell transdifferentiation contribute to PNEC hyperplasia in WT mice following allergen challenge. (**A**) Representative images of WT lung sections stained with anti-CGRP antibody. (**B** and **C**) Quantification of PNEC numbers based on CGRP staining or *Ascl1*-lineage. One-way ANOVA was used (*n* = 3–8 for each group). (**D**) Representative images of *Ascl1*-lineaged lung sections exposed to saline or HDM stained with anti-Ki67 antibody to detect proliferation in PNEC clusters on top row. Arrowheads point to proliferating PNECs on top row. Representative images of *Scgb1a1*-lineaged lung sections exposed to saline or HDM stained with anti-CGRP antibody to trace club cell transdifferentiation to PNECs on bottom row. Arrowheads point to transdifferentiating PNECs on bottom row. (**E**) Quantification of *Ascl1*-lineage overlap with Ki67^+^ cells over the total of *Ascl1*-lineaged cells. Student’s *t* test was used (*n* = 3 for each group). (**F**) Quantification of *Scgb1a1*-lineage overlap with CGRP^+^ cells over the total of CGRP^+^ cells. Student’s *t* test was used (*n* = 3, 4 for each group). (**G**) Representative images of *Ascl1* lineage lung sections exposed to saline or HDM stained with anti-CGRP antibody. (**H**) Representative images of protruding NEBs marked by anti-CGRP antibody in saline or HDM-challenged lung sections. Asterisks represent gap between epithelial layer and the subjacent mesenchymal cells. (**I**) Quantification of protruding NEBs over the total number of PNEC clusters. One-way ANOVA was used (*n* = 3–5 for each group). Each data point represents the average value of ~25 sections from 1 lung. ** for *P* < 0.01, *** for *P* < 0.001, and **** for *P* < 0.0001. Error bars represent mean ± SD. Arrowheads point to PNECs with elongated morphology following allergen challenge. Scale bar size 50 μm.

PNECs are increased in number in asthmatic patient lungs, which may contribute to their exacerbated response to allergen (3). To address the mechanism underlying such PNEC hyperplasia, we exposed WT adult mice to varying doses of HDM and quantified the number of PNECs. We found a statistically significant increase in the number of CGRP^+^ PNECs starting after the first dose, and a similar extent of increase was sustained following repeated doses (Figure 3A and Supplemental Figure 8). After the standard regimen of 4 HDM doses, there was an approximately 2.09-fold increase of total CGRP^+^ PNECs (saline control mean ± SD 28.73 cells ± 11.672, *n* = 6 mice; HDM 60.11 cells ± 19.702, *n* = 9 mice, *P* = 0.0033). Both cluster number and cells per cluster were increased to contribute to the rise in total PNECs (Figure 3B).

To address whether excess PNECs arose from existing PNECs, we subjected *Ascl1^creERT2^ Rosa26^reporter^* mice to HDM challenge. There was an approximately 1.34-fold trending increase of *Ascl1*-lineaged cells following HDM challenge (saline control 50.01 cells ± 10.725, *n* = 4 mice; HDM 66.87 cells ± 6.524, *n* = 5 mice, *P* = 0.1422) (Figure 3C and Supplemental Figure 8C). The increase was found in both solitary cells and large (5+) clusters (Supplemental Figure 9, B and C). There was also a trending increase of Ki67^+^ *Ascl1*-lineaged cells as quantified by sample (saline control 0.495% ± 0.070, *n* = 3 mice; HDM 4.9815% ± 2.574, *n* = 3 mice), and this increase was significant when quantified by section (saline control 0.990% ± 0.200, *n* = 48 sections; HDM 4.516% ± 4.440, *n* = 48 sections) (Figure 3, D and E, and Supplemental Figure 10B). This is consistent with the notion that elevated proliferation of existing PNECs contributes to PNEC hyperplasia. However, the more robust increase in overall CGRP^+^ PNEC number suggested that proliferation of existing cells was not the only contributor. In support of this, *Ascl1^creERT2^ Rosa26^reporter^* mice exposed to HDM showed CGRP^+^ PNECs that are not lineaged (Supplemental Figure 10A). In extreme examples, there were clustered NEBs at the airway branchpoint junction that were composed of a large number of non-*Ascl1*-lineaged cells (Supplemental Figure 10A). Comparatively, there was no change in or even reduced number of PNECs in the trachea (Supplemental Figure 11). These results suggest that in normal lung when exposed to HDM, transdifferentiation of non-PNECs into PNECs also contributes to PNEC hyperplasia.

### Club cells transdifferentiate into PNECs following allergen exposure.

To address the source of the transdifferentiated PNECs in the lung, we focused on nearby club cells as they are known to have progenitor capacity. We lineaged club cells in *Scgb1a1^creERT2^ Rosa26^tdTomato^* mice and then exposed them to HDM. We note that in saline control, aside from club cells, this reporter also sporadically labeled approximately 7.67% ± 6.95 (*n* = 4) of PNECs (Figure 3C). Following HDM challenge, the overlap increased to approximately 21.11% ± 4.66 (*n* = 4), suggesting increased contribution from club cells (Figure 3F and Supplemental Figure 10C). Consistent with this, in *Scgb1a1^creERT2^ Ascl1^fl/fl^* mutants, PNEC hyperplasia was blunted, likely because in the absence of *Ascl1*, club cells could no longer transdifferentiate into PNECs following HDM (Supplemental Figure 12). In comparison, exposure of basal cell reporter mice (*Tp63^creERT2^ Rosa26^tdTomato^*) to HDM did not lead to labeling of PNECs, suggesting that basal cells do not transdifferentiate into PNECs (Supplemental Figure 13). Despite the lack of contribution to PNECs, there was an unexpected increase of basal-lineaged cells in the intrapulmonary airway following HDM (Supplemental Figure 13). Similarly, basal-lineaged cells expanded into luminal cells in the trachea following HDM. These findings raised the possibility that HDM induces an airway injury response, leading to basal cell–originated differentiation into non-PNEC luminal cell types. Taken together, these data suggest that proliferation of existing PNECs and transdifferentiation of club cells contribute to allergen-induced PNEC hyperplasia.

In mice challenged with HDM, with the increase in the number of PNECs per cluster, we observed an increased number of cells exhibiting elongated morphology within NEBs following HDM challenge (Figure 3G). Concomitant with these changes, NEBs bulged into the lumen (Figure 3H). The proportion of NEBs with such protruding morphology increased from approximately 2.00% ± 0.822 at baseline to approximately 13.98% ± 3.13 after the first dose of HDM and was then maintained at approximately 8% of all NEBs (Figure 3I).

Aside from PNECs, there appeared to be a gradual increase of goblet cells with subsequent HDM challenges, based on AGR2, MUC5B, and MUC5AC staining (Supplemental Figure 14). In contrast, E-cadherin expression along the airway and within PNEC clusters increased after the first dose but was reduced following subsequent challenges (Supplemental Figure 14). Taken together, allergen challenge led to airway remodeling, including increased PNECs and goblet cells, altered PNEC morphology, and disrupted adherens junction.

### Single-cell transcriptomic profiling of PNECs reveals heterogeneity.

**Figure 4:**
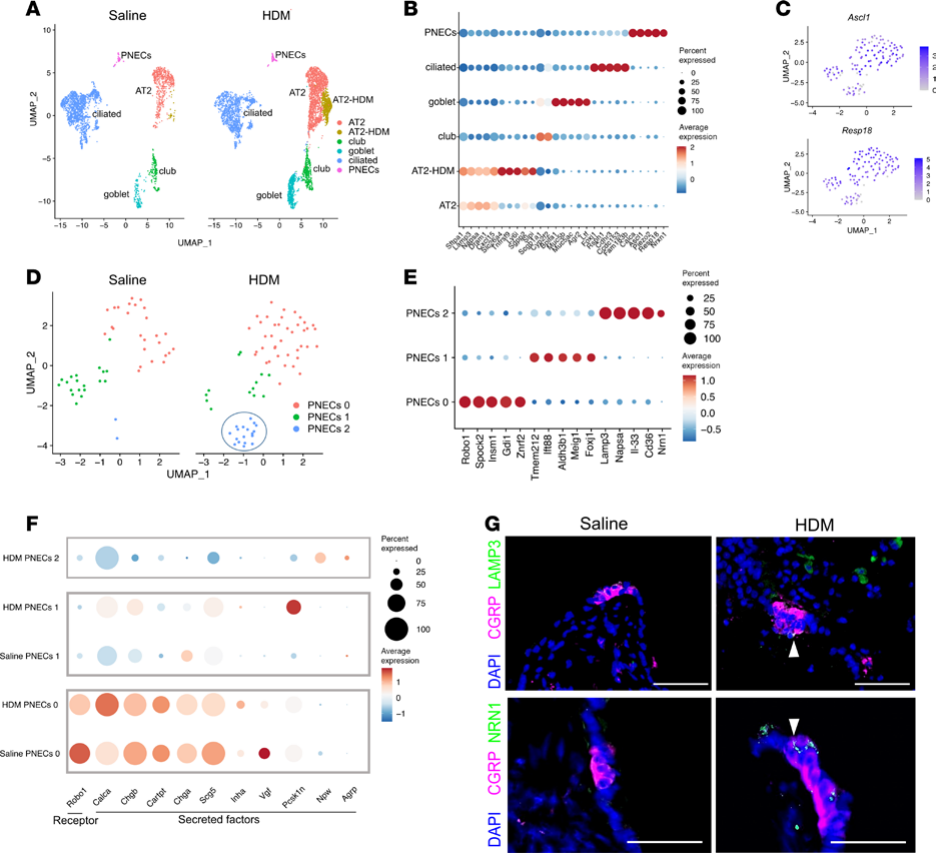
Single-cell transcriptomic characterization of epithelial cells with or without allergen challenge. (**A**) Uniform manifold approximation and projection (UMAP) plot of integrated scRNA-Seq data of *Ascl1*-lineaged cells supplemented with EpCAM^+^ epithelial cells. (**B**) Dot plot showing top marker genes for each epithelial population. (**C**) Feature plot of PNEC markers. (**D**) UMAP plot of PNEC populations. (**E**) Dot plot showing top marker genes for PNEC clusters. (**F**) Dot plot showing cell surface or secreted factor genes for PNEC clusters with or without HDM. Cluster 2 is primarily present in the HDM group. (**G**) Representative images of WT lung sections stained with anti-CGRP and either anti-LAMP3 or anti–Neuritin-1 (anti-NRN1) antibodies, as indicated. Arrowheads point to cells with coexpression. Scale bar size 50 μm.

To address how allergen exposure alters the transcriptome of lung epithelial cells including PNECs, we performed scRNA-Seq using PNECs sorted from *Ascl1^creERT2^* *Rosa26^tdTomato^* lungs, supplemented with EpCAM^+^ epithelial cells from lungs following either the fourth HDM or saline exposure (Supplemental Figure 15). After quality control and filtering, 2,689 saline and 4,022 HDM cells were integrated using Seurat pipeline (15, 16). Five populations (AT2, club, goblet, ciliated, and PNECs) were shared between saline and control, and a distinct population of AT2 cells was found in HDM-challenged lung (Figure 4, A and B, and Supplemental Figure 16).

For this study, we focused on PNECs. In silico, we isolated just the integrated PNECs, all expressing canonical PNEC markers, including *Ascl1* and *Resp18* (Figure 4C). Subclustering of these cells led to 3 subgroups where 1 cluster (cluster 2) was primarily present in the HDM lung (Figure 4D). Marker analysis revealed distinct markers for each of the PNEC clusters. For cluster 0, a top marker is roundabout guidance receptor 1 (*Robo1*), which we have previously shown to be essential for PNEC clustering (17). RNAscope showed that in the control lung, *Robo1* was expressed primarily in clustered PNECs in NEBs compared with solitary PNECs (Supplemental Figure 17). Following HDM, *Robo1* expression was reduced in isolated PNECs in some of the NEBs (Figure 4, E and F, and Supplemental Figure 18A). This is consistent with the observations of reduced E-cadherin (Supplemental Figure 14), suggestive of cells breaking away from NEB aggregates following allergen challenge. For cluster 1 PNECs compared with other PNECs, multiple top markers were intriguingly enriched in ciliated cells, such as *Foxj1* (Figure 4, E and F). Such expression of FOXJ1 in a small subset of PNECs was verified by staining (Supplemental Figure 18A). For cluster 2, which was primarily found in HDM-challenged lungs, multiple top markers were also enriched in angiotensin II receptor type 2 (AT2) cells outside of PNECs, such as lysosomal associated membrane protein 3 (*Lamp3*) and *Il33* (Figure 4G and Supplemental Figure 18B). Staining verified colocalization of CGRP with either LAMP3 or *Il33* in both clustered and solitary PNECs (Figure 4G and Supplemental Figures 18 and 19). Such expression suggests that these PNECs have adopted some AT2-like characteristics, such as immune surveillance, protein cleavage, and processing in preparation for secretion.

Another cluster 2 marker, *Neuritin-1* (NRN1), is affiliated with neuronal elongation. By antibody staining, NRN1 was also found to colocalize at the apical end of the spindly PNECs that we have observed, potentially contributing to the elongated morphology (Figure 3G and Figure 4G). Given that one of the key functions of PNECs is to secrete bioactive signals, we compared their expression in PNEC clusters in saline versus HDM. We found that some were increased, some remained the same, and some were decreased (Figure 4F). For example, *Calca*, encoding CGRP, was increased in PNECs, consistent with our prior finding (3).

### Downregulation of Notch signaling contributes to PNEC hyperplasia following allergen challenge.

**Figure 5:**
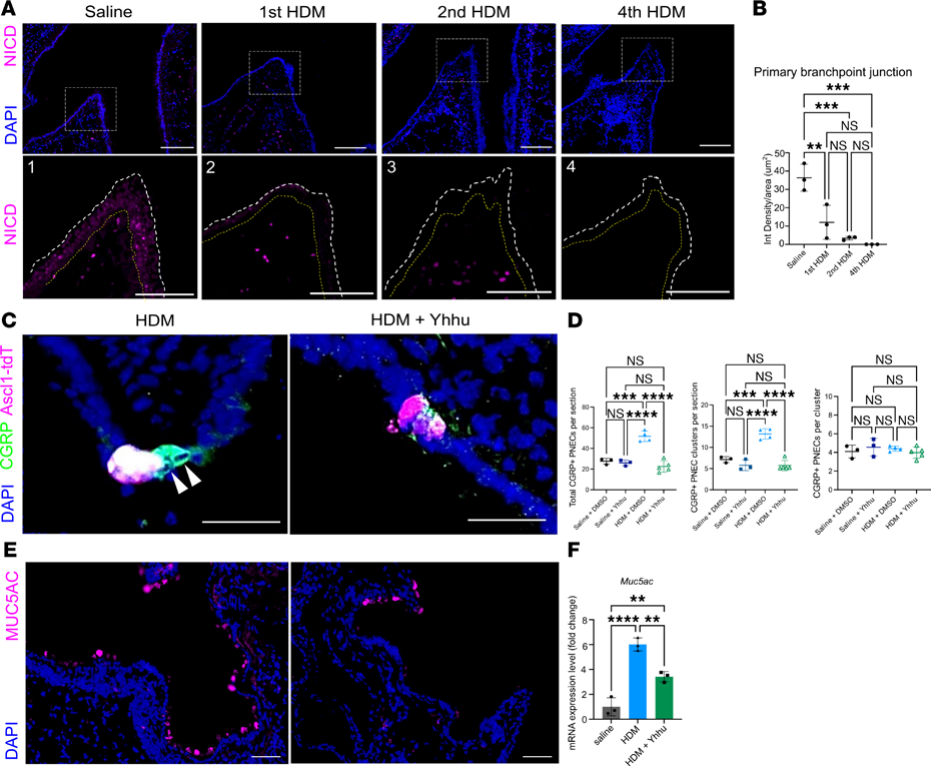
Downregulation of Notch signaling pathway contributes to PNEC increase following allergen challenge. (**A**) Representative images of WT lung sections stained with anti-NICD antibody. Dashed lines delineate the airway epithelium. (**B**) Quantification of intensity density (sum of pixels) over the area of traced epithelial layer (Supplemental Figure 18A). (**C**) Representative images of *Ascl1*-lineaged lung sections exposed to HDM or HDM with *Notch1* agonist, Yhhu 3972, stained with anti-CGRP antibody. Arrowheads point to PNECs that are not *Ascl1*-lineage. (**D**) Quantification of PNEC numbers based on CGRP staining. (**E**) Representative images of lung sections exposed to HDM or HDM with *Notch1* agonist, Yhhu 3972, stained with anti-MUC5AC antibody. (**F**) qRT-PCR of whole lungs under noted conditions. One-way ANOVA was used (*n* = 3–5 for each group). Each data point represents the average value of ~25 sections from 1 lung in **D**. * for *P* < 0.05, ** for *P* < 0.01, *** for *P* < 0.001, and **** for *P* < 0.0001. Error bars represent mean ± SD. Scale bar size 50 μm.

To address the molecular mechanism underlying increased PNECs, we focused on the Notch signaling pathway because it is a key regulator of *Ascl1* (6, 12), which is essential for PNEC formation (3, 6) and maintenance (Figure 1). To determine if altered Notch signaling could mediate allergen-induced PNEC increase, we assessed the expression level of the Notch intracellular domain (NICD), a product of proteolytic events following pathway activation and a central effector of Notch signaling (18). At the primary branchpoint junctions where clustered PNECs are enriched, NICD was consistently detected, albeit at a low level at baseline (Figure 5A). Starting after the first dose of HDM and with each HDM challenge, NICD level was reduced not only in the branchpoint junction but also in the primary bronchus (Figure 5, A and B, and Supplemental Figure 19, B and C). In comparison, in the lateral and posterior distal airways, NICD level was undetectable at baseline, increased after the first and second doses of HDM, and reduced back to undetectable by the fourth dose (Supplemental Figure 19, B and C).

To uncover if decreased Notch signaling plays a role in PNEC hyperplasia, we utilized a Notch agonist, Yhhu 3792, which has been reported to upregulate *Notch1* levels based on in vitro experiments (19). Mice were subjected to HDM challenges with or without intranasal administration of Yhhu 3792. After 2 doses, by either *Ascl1*-lineage analysis or CGRP staining, PNEC hyperplasia did not occur following HDM with Yhhu 3792 (Figure 5C and Supplemental Figure 19E). All CGRP^+^ PNECs colocalized with *Ascl1*-lineaged cells in HDM-challenged mice in the presence of Yhhu 3792 (Figure 5C and Supplemental Figure 19). Not only did the agonist prevent PNEC formation, but it also reduced CGRP expression in PNEC clusters (Figure 5, C and D). Moreover, goblet cell metaplasia following allergen challenge was also acutely reduced in the presence of the Notch agonist shortly after treatment (Figure 5, E and F). To address if changes in Notch signaling also contribute to PNEC reemergence in the *Ascl1* mutant, we treated *Ascl1^cre/fl^* mutant and controls to HDM with Yhhu 3792. Ectopic PNECs failed to emerge in the *Ascl1* mutant (Supplemental Figure 19F). These data together suggest that downregulation of Notch signaling contributes to PNEC increase following allergen challenge.

## Discussion

Following the discovery that PNECs, despite their rarity, play an essential role in amplifying asthmatic response (3), here we show that PNEC dynamics is an important feature of the condition. In WT mice, PNECs increase in number following HDM challenge because of both proliferation of existing PNECs and club cell transdifferentiation. We found that altered Notch signaling is responsible for such an increase in PNEC number. Single-cell transcriptomic profiling of PNECs demonstrated that aside from increased production of neuropeptides and neurotransmitters, an allergen-specific population emerges. These results provide insight into how PNECs are sentinels that modulate lung response to the environment.

Building on knowledge that *Ascl1* is essential for the formation of PNECs, our findings here demonstrate that it is also required for their maintenance. PNECs have been shown to be the cell of origin of small cell lung cancer (SCLC) (20–25). Inactivation of *Ascl1* in SCLC cell lines or in mouse SCLC models showed reduced tumorigenesis (26, 27), suggesting that aside from our findings in normal PNECs, *Ascl1* is also required for the maintenance of SCLC cells. In normal lungs, we show that inactivation of *Ascl1* led first to reduced level of neuroendocrine markers, followed by apoptosis. ChIP-Seq data showed that the cardinal antiapoptotic factor, B cell CLL/lymphoma 2 (BCL2), is a direct downstream target of ASCL1, and knockdown of *Ascl1* led to reduced *Bcl2* mRNA and protein levels (28). These in vitro data suggest a possible mechanism where inactivation of *Ascl1* may lead to PNEC apoptosis via downregulation of *Bcl2*.

In our previous study utilizing the *Shh^cre^ Ascl1^fl/fl^* mutants, allergen challenge did not lead to the reappearance of PNECs (3). This is because *Ascl1* is inactivated in all lung epithelial cells, and *Ascl1* is essential for transdifferentiaton of non-PNECs into PNECs. In the absence of PNECs, the *Shh^cre^ Ascl1^fl/fl^* mutant mice displayed significantly reduced type 2 immune recruitment and goblet cell metaplasia, allowing us to dissect how PNECs act through neuropeptides and neurotransmitters to control these asthmatic responses (3). In the current study, utilizing the *Ascl1^cre/fl^* mutants where *Ascl1* is inactivated specifically in PNECs, allergen challenge led to the spontaneous reappearance of PNECs (Figure 2). The presence of an intact *Ascl1* gene in the remainder of the airway epithelial cells, particularly in club cells, allowed these cells to transdifferentiate into PNECs. These PNECs that arose from transdifferentiation were capable of facilitating goblet cell metaplasia as well as AHR and type 2 immune response following allergen challenge, similar to controls.

Following allergen challenge, while *Ascl1^cre/fl^* mutants recovered their PNECs primarily through transdifferentiation, in WT mice, PNEC hyperplasia occurred from both club cell transdifferentiation and proliferation of existing PNECs. Furthermore, the proportion of PNECs expressing key neuropeptides, such as CGRP, increased following allergen challenge, particularly in solitary cells (Supplemental Figure 9, A and B), suggesting induction of neuropeptide expression by allergen in addition to the induction of PNECs. It is likely that all these allergen-induced PNEC changes contribute to the exacerbated allergen response.

It is interesting that PNEC hyperplasia is already apparent after the first dose of HDM, suggesting that it is an early response (Figure 3, A and B). Furthermore, PNEC number is only minimally further elevated with subsequent doses. Such a plateau could be because once ectopic PNECs emerge from transdifferentiation, they express Notch ligands such as DLL1, which could act on adjacent cells to upregulate Notch signaling and thereby prevent them from becoming more PNECs (29, 30). Compared with these fast-to-rise and fast-to-plateau dynamics, type 2 immune cell number and goblet cell gene expression steadily increased after each dose (4).

Notch pathway activation in PNECs has been shown to induce cell proliferation and their transdifferentiation toward other luminal cell types, such as club cells (23, 31). Conversely, in the current study, following allergen challenge, club cells transdifferentiated into PNECs instead. Previous studies have shown conflicting results on Notch upregulation versus downregulation following allergen challenge by total protein quantification and transcriptional analyses (32–35). We show here that by focusing specifically on the proximal airway epithelium where PNECs are enriched, NICD is reduced with increasing doses of HDM (Figure 5, A and B, and Supplemental Figure 18). In distal regions, NICD initially increased with HDM and reduced over time (Supplemental Figure 18). Administration of Notch agonist in HDM-treated mice reversed PNEC hyperplasia, suggesting that the HDM-induced reduction of Notch signaling, acting on club cells, contributed to club cell transdifferentiation into PNECs (Figure 5, C and D). Additionally, we observed reduced goblet cell metaplasia in lungs collected within 24 hours following 2 doses of HDM and Notch agonist administration, suggesting a short-term effect of Notch activation (Figure 5, E and F).

At the cellular level, PNEC diversity is reflected by the presence of both clustered and solitary cells. While solitary PNECs are dispersed across the airway epithelium, a salient feature of NEBs is their enrichment at branchpoint junctions, where inhaled particles are expected to concentrate. Previous work from our laboratory showed that the expression of *Robo* genes in PNECs is essential for cluster formation (17). In our scRNA-Seq data, *Robo1* is expressed at a higher level in PNEC cluster 0 compared with the remaining clusters (Figure 4E). Our staining data validated that *Robo1*-expressing cluster 0 PNECs are clustered in NEBs, while cluster 1 PNECs are solitary (Supplemental Figure 17).

PNEC cluster 2, uniquely found in HDM-challenged lungs, express genes such as *Il-33*, a critical upstream alarmin in type 2 immune response, as well as *Chia1*, *Chil1*, and *Chil4* (Supplemental Figure 17), proteases involved in the degradation of chitin, an allergenic component of allergens such as HDM (36, 37). In addition to the enzymatic activity of chitinases, chitinase-like molecules have been demonstrated to bind to the IL-13 receptor, IL13α2, and mediate Th2-mediated allergen immune response (38). The expression of chitinases and chitinase-like molecules in HDM-induced PNECs suggests a potentially novel mechanism of how these PNECs could contribute to allergen-induced response.

The findings in this study together demonstrate that PNECs are a heterogeneous population that also undergo dynamic changes in disease settings, such as in asthma. PNEC hyperplasia is also found in human patients with asthma and likely contributes to chronically exacerbated airway response to allergen (3). Our findings here revealed the mechanism underlying allergen-induced PNEC hyperplasia and could inform options to control asthmatic responses via modulating PNEC number and function.

## Methods

### Sex as a biological variable.

Male mice were specifically used when studying response to HDM as previous studies in our lab established that there was variability in brain activity and respiratory response based on flexiVent measurements in female mice (4). In establishing the *Ascl1*-mutant model and collecting epithelial cells for scRNA-Seq, equal numbers of male and female mice were studied. Similar findings were found for both sexes.

### Animals.

Mouse lines used were tamoxifen-inducible, knockin Cre recombinase drivers *Ascl1^creERT2^* (39), *Scgb1a1^creERT2^* (40), and *Tp63^creERT2^* and constitutively active reporter *Rosa26^ZsGreen^* (41) and *Rosa26^tdTomato^* (41). Mice were purchased from Jackson Laboratory, with the exception of *Ascl1^fl^* (42) mice that were gifts from Francois Guillemot’s lab (Francis Crick Institute, London, United Kingdom) and *Shh^cre^* mice from Clifford J. Tabin’s lab (Harvard Medical School, Boston, Masschusetts, USA). Genotyping was performed on ear clips utilizing oligonucleotide primers previously reported for each strain. Early postnatal stage and adult mice aged 2–3 months were used in all experiments, with similar numbers of males and females. Mice were maintained in 12-hour light/12-hour dark cycle with food and water provided ad libitum. All mice were housed in American Association for Accreditation of Laboratory Animal Care–accredited facilities and labs at UCSD. All animal husbandry, maintenance, and experiments were performed in accordance with UCSD’s IACUC-approved protocols.

### Tamoxifen induction of Cre recombination.

Tamoxifen stock solution (10 mg/mL, MilliporeSigma) was prepared by sonication in corn oil and stored at –20°C. Intraperitoneal injections at the dose of 25 mg/kg were administered every other day for the periods indicated in the figure scheme for the *Ascl1* mutant (Figure 1A). In remaining reporter mice, adult mice (at 8 weeks old) were injected with tamoxifen twice within 48 hours to induce cre for the activation of the reporter.

### Allergen challenge with HDM and Notch agonist.

HDM (*Dermatophagoides pteronyssinus*, Greer Labs) stock solution (5 mg/mL) was prepared in saline solution and stored at –20°C. Mice were anesthetized with isoflurane prior to nasal administration of 50 μg (dissolved in 20 μL of saline) of HDM extract. HDM in adult mice (at 8 weeks old) was administered on days 0, 7, 14, and 21 (Figure 2A). For controls, 20 μL of saline was administered intranasally to adult mice in the same regimen. All mice were euthanized under IACUC guidelines 24 hours following the last HDM challenge for analysis.

For Notch agonist, Yhhu 3792 (Tocris 6599) powder was ground with 0.5% DMSO and 1% Tween and diluted into suspension with saline to approximately 50 μg. Yhhu 3792 was administered intranasally following HDM; 0.5% DMSO and 1% Tween was administered as the vehicle group without Yhhu 3972.

### Immunohistochemistry.

Mice were euthanized by CO_2_ asphyxiation. For cryosections, lungs were immediately dissected following inflation at 35 cm H_2_O airway pressure with 4% (v/v) paraformaldehyde (PFA; Electron Microscopy Sciences), prepared fresh by dilution of a 32% stock solution in phosphate-buffered saline (PBS). Inflated lungs were fixed overnight at 4°C and dehydrated in 30% (w/v) sucrose in PBS overnight before embedding in optimum cutting temperature (O.C.T.; Sakura) compound and storage at –80°C. For flash-frozen sections, lungs were inflated with 20% O.C.T. compound and immediately embedded in O.C.T. compound before flash-freezing in liquid nitrogen. Tracheal samples were dissected and mounted similarly, without inflation.

Coronal sections (10 μm and 30 μm) were prepared using a cryostat, and cryosections were mounted to Superfrost Plus slides (Fisherbrand, Thermo Fisher Scientific). Sections were dried at room temperature for 15–60 minutes before washing twice for 5 minutes with PBS, postfixing with 4% PFA, and washing twice for 5 minutes with 0.1% (v/v) Tween 20 in PBS. Sections were incubated for 1.5 hours at room temperature in 5% (v/v) normal goat serum in 0.1% Tween 20 with PBS (blocking solution), then incubated overnight at 4°C with primary antibodies in blocking solution in a humid chamber. The following day, sections were washed 3 times for 10 minutes each in 0.1% Tween 20 PBS, then incubated for 1 hour at room temperature in blocking solution containing Alexa Fluor–conjugated (AF-conjugated) secondary antibodies and DAPI. Stained sections were finally washed 3 times for 5 minutes each in 0.1% Tween 20 PBS and mounted on coverslips with Fluoromount-G (SouthernBiotech) as the mounting medium. Mounted specimens were stored at 4°C until microscopy (ZEISS Axio Imager 2).

The following primary antibodies were used: mouse anti-MUC5AC monoclonal antibody (5 mg/mL, MilliporeSigma MAB2011), mouse anti-CGRP (human) monoclonal antibody (2 mg/mL, Lifespan Biosciences ab81887), mouse anti-TUJ1 monoclonal antibody (1 mg/mL, MilliporeSigma BAM1195), mouse anti-FOXJ1 monoclonal antibody (0.5 mg/mL, Invitrogen 5013931), rabbit anti-CGRP polyclonal antibody (2 mg/mL, MilliporeSigma C8198), rabbit anti-SYP polyclonal antibody (5 mg/mL, Thermo Fisher Scientific RB-1461-P1), rabbit anti-SCGB1A1 polyclonal antibody (5 mg/mL, Seven Hills Bioreagents WRAB-3950), rabbit anti-MUC5B polyclonal antibody (0.23 mg/mL, Cloud Clone Corp PAA684Mu01), rabbit anti-AGR2 monoclonal antibody (1 mg/mL, Cell Signaling Technology 130625), rabbit anti-LAMP3 monoclonal antibody (1 mg/mL, Synaptic Systems 391 005), rabbit anti-NRN1 polyclonal antibody (1 mg/mL, Invitrogen PA5-20368), and guinea pig anti-SYT1 monoclonal antibody (1 mg/mL, Synaptic Systems 105 318). The following secondary antibodies were used: Cy3-conjugated goat anti-mouse IgG (2 mg/mL, Jackson ImmunoResearch Labs 2338680), Cy5-conjugated goat anti-mouse AffiniPure IgG (2 mg/mL, Jackson ImmunoResearch Labs 2338714), FITC-conjugated goat anti-rabbit (2 mg/mL, Jackson ImmunoResearch Labs 14165), Cy3-conjugated goat anti-rabbit IgG (2 mg/mL, Jackson ImmunoResearch Labs 2338000), and AF488-conjugated goat anti–guinea pig (2 mg/mL, Invitrogen 2337415).

For NICD immunostaining of cryosections, tyramide signal amplification was performed using the detection kit (Invitrogen). Sections were stained with rabbit anti-NICD primary antibody (2 mg/mL, Cell Signaling Technology 4147S), washed, and incubated with goat anti-rabbit biotin secondary (Invitrogen 65-6140) followed by incubation with horseradish peroxidase streptavidin. Following washes, the sections underwent incubation with AF594-conjugated tyramide (prepared according to manufacturer’s instructions) (Invitrogen B40925) for 5 minutes at room temperature. Slides were then washed, mounted, and imaged.

### RNAscope.

Adult fresh-frozen sections from control and HDM-challenged mice were stained with probes from mouse Robo1 (ACD 475951-C1) and Il-33 (ACD 400591-C2) using the RNAscope Multiplex Fluoromount Reagent Kit v2 (ACD 323100). Staining was performed using manufacturer’s instructions for fresh-frozen sections. Sections were counterstained with CGRP antibody to detect the presence of PNECs.

### Microscopy and imaging.

Cryosections (10–30 μm) were imaged using a ZEISS Axio Imager 2/LSM 880 at 5×, 10×, 20×, or 40× objective. Remaining images were taken with ZEISS laser scanning confocal microscope with an inverted 40× or 63× oil immersion objective. Sections were selected to best match the representative field and to cover different depths, if necessary.

### Transcriptional analysis (qRT-PCR).

Dissected lungs were stored in TRIzol (Invitrogen) at –80°C until further processing. Total RNA was extracted using instructions from the RNeasy Mini RNA extraction kit (QIAGEN). cDNA was generated using the iScript Select cDNA Synthesis Kit (Bio-Rad). qRT-PCRs were performed using gene-specific primers and iTaq SYBR Green (Bio-Rad) by CFX Connect system (Bio-Rad). At least 3 technical and 3 biological replicates were performed for each gene, if not otherwise noted. Standard quantified values were normalized to the β-actin housekeeping genes, and relative changes in gene expression were calculated using the ΔΔCT method. All primer sequences used for qRT-PCR analysis are listed: Calca forward: 5′ CCTTTCCTGGTTGTCAGCATCTTG 3′, Calca reverse: 5′ CTGGGCTGCTTTCCAAGATTGAC 3′, Ascl1 forward: 5′ CTCCCCATTTGACGTCGTTG 3′, Ascl1 reverse: 5′ CTACGACCCTCTTAGCCCAG 3′, Tac1 forward: 5′ GGTCCGACAGTGACCAGATCAAG 3′, Tac1 reverse: 5′ AAAGAACTGCTGAGGCTTGGGTC 3′, Muc5ac forward: 5′ TGACTCATCTGCGTGCCTT 3′, Muc5ac reverse: 5′ AGGCCTTCTTTTGGCAAGGTT 3′, Scgb1a1 forward: 5′ ATGAAGATCGCCATCACAATCAC 3′, Scgb1a1 reverse: 5′ GGATGCCACATAACCAGACTCT 3′, Foxj1 forward: 5′ CTCCTATGCCACTCTCATCTGC 3′, Foxj1 reverse: 5′ GACAGGTTGTGGCGGATGGAAT 3′, β-actin forward: 5′ CGGCCAGGTCATCACTATTGGCAAC 3′, and β-actin reverse: 5′ GCCACAGGATTCCATACCCAAGAAG 3′.

### Tissue processing and flow cytometry.

Mice were anesthetized and administered an intravenous dose of AF700-conjugated CD45 (BioLegend, no. 103128; 10 μg per mouse) to discriminate between circulating and lung-resident immune cells. Five minutes later, the mice were euthanized, and their lungs were dissected. Each lung was mechanically dissociated in GentleMACS C tubes (Miltenyi Biotec) containing 5 mL RPMI 1640 (Thermo Fisher Scientific) supplemented with 10% fetal bovine serum (FBS), 1 mM HEPES (Life Technologies), 1 mM MgCl_2_ (Life Technologies), 1 mM CaCl_2_ (MilliporeSigma), 0.525 mg/mL collagenase/dispase (Roche), and 0.25 mg DNase I (Roche). The GentleMACS “mouse lung 1-2” program (Miltenyi Biotec) was used for initial dissociation. Samples were then incubated at 37°C with shaking (~150 rpm) for 30 minutes. Following incubation, lungs were further dissociated using the GentleMACS “mouse lung 2-1” program and strained through a 70 μm filter (Miltenyi Biotec). Red blood cells (RBC) were lysed by adding 1 mL of RBC lysis buffer (BioLegend) for 1 minute at room temperature. The remaining cells were pelleted (1,500 rpm, 4°C, 5 minutes), counted with a hemocytometer (Thermo Fisher Scientific), and diluted to approximately 1 × 10^6^ cells/mL. Cells were treated with Fc-block (5 mg/mL, BD Biosciences) before staining with a cocktail of surface marker antibodies.

For lung myeloid cells, the following antibodies were used: 1:100 BV605-conjugated anti-F4/80 (BioLegend, no. 123133), 1:500 BV510-conjugated anti-CD45 (BioLegend, no. 110741), 1:1,000 APC-conjugated anti-CD11c (BioLegend, no. 117310), 1:1,000 PE-Cy7-conjugated anti-Ly6G (BioLegend, no. 560601), and 1:2,000 PE-CF594-conjugated anti-CD11b (BioLegend, no. 101256). For lung lymphoid cells, the following antibodies were used: 1:200 FITC-conjugated anti-CD45 (BioLegend, no. 103108), 1:100 APC-Cy7-conjugated anti–IL-7Ra (BioLegend, no. 135040), 1:200 V450-conjugated lineage mix (anti-CD19 [TONBO, no. 50-201-4944], anti-CD11c [TONBO, no. 50-201-4937], anti-F4/80 [TONBO, no. 50-201-4978], anti-NK1.1 [BD Biosciences, no. 560524], anti-TER119 [BD Biosciences, no. 560504], anti-TCR γδ [Invitrogen, no. 48-5711-82]), 1:100 BV510-conjugated anti-ST2 (BD Biosciences, no. 745080), 1:200 PE-Cy7-conjugated anti–TCR-β (BioLegend, no. 109222), 1:100 BV604-conjugated anti-CD4 (BioLegend, no. 100548), anti–cleaved caspase-3 (Cell Signaling Technology, Asp175, no. 9661), anti-Ki67 (Cell Signaling Technology D3B5, no. 9129), and 1:2,000 PerCP-Cy5.5-conjugated anti-CD90.2 (BioLegend, no. 105338). Cells were labeled with live/dead dye (Ghost Dye Red 780, TONBO, no. 13-0865-T100 or Ghost Dye Violet 450, TONBO, no. 13-0863-T100), then fixed using BD Biosciences Stabilizing Fixative. Samples were analyzed using a BD Biosciences FACSCanto RUO – ORANGE flow cytometer (405, 488, and 640 nm lasers) at the VA San Diego Health Care System Flow Cytometry Core. Data were further analyzed and visualized with FlowJo (Tree Star). Eosinophils, ILC2s, and Th2 cells were gated on live, resident CD45^+^ singlets.

### Airway hyperreactivity assayed by flexiVent.

For AHR measurements, mice were anesthetized and given acepromazine (10 mg/kg, intraperitoneal). They were then tracheotomized with a 20 G sterile catheter, connected to a flexiVent pulmonary mechanics apparatus (SCIREQ), and ventilated at a tidal volume of 9 mL/kg with a frequency of 150 breaths/min. The weight of each mouse was entered into flexiVent at the beginning of each measurement series. Positive end-expiratory pressure was maintained at 300 mm H_2_O. A 10-second nebulization was performed for each methacholine (Mch) dose (0, 6, 12, or 24 mg/mL in 0.9% NaCl). The resistance (Rrs) and elastance (Ers) of the respiratory system were determined in response to each Mch challenge, and the mean maximal Rrs and Ers across 12 measurements per dose were calculated. Statistical comparisons at each Mch concentration were carried out separately.

### PNEC and epithelial cell enrichment by FACS.

To enrich live PNECs for scRNA-Seq profiling, adult *Ascl1^creERT2^ Rosa26^tdTomato/+^* mice (roughly 2- to 3-month old male and female littermates) were administered 2 doses of i.p. injection of tamoxifen at P12 and P14, then underwent HDM regimen over the course of 4 weeks from 8 weeks old. Mice were euthanized by CO_2_ asphyxiation 24 hours following the last HDM challenge and perfused with approximately 10 mL of PBS into the right cardiac ventricle.

Lungs were dissected and pooled (6 lungs per collection round) to ensure sufficient numbers of labeled PNECs for sorting. Whole lungs, excluding the trachea, were dissociated by cutting them into small pieces with surgical scissors, then enzymatically digested in RPMI containing 1 mg/mL collagenase type 4 (Roche), 5 U/mL dispase (Thermo Fisher Scientific), and 20 μg/mL DNase I (Roche) for 30 minutes at 37°C in a bacterial shaker. Following digestion, lung tissue was pressed through a 70 mm cell strainer, and enzymes were quenched with an excess volume of RPMI containing 10% (v/v) FBS. Samples were spun down at 1,500 rpm for 5 minutes at 4°C and incubated in RBC lysis buffer (BioLegend) for 3 minutes on ice. Five-minute spins continued to be performed between each step, and cell-free supernatants were removed through vacuum aspiration. RBC-lysed cell suspensions were depleted for endothelial cells and leukocytes using anti-CD31– (0.5 mg/mL, BioLegend, 102407) and anti-CD45– (0.5 mg/mL, BioLegend, 103102) coated magnetic microbeads and MACS separation columns, according to the manufacturer’s instructions (EasySep, STEMCELL Technologies). PBS with 2% FBS (FACS buffer) was used as the suspension solution for magnetic labeling and for cell application onto columns. Endothelial/leukocyte-depleted cell suspensions were stained with allophycocyanin-conjugated (APC-conjugated) anti-CD326 (EpCAM G8.8, 0.5 mg/mL, Invitrogen) at 1:200 dilution in FACS buffer. Stained cell suspensions were washed twice for 5 minutes each in FACS buffer to remove residual unbound antibodies and resuspended in FACS buffer containing 1 μg/mL DAPI as a live-dead cell indicator before proceeding to FACS.

Cell sorting was performed in the Sanford Consortium FACS facility using a BD Biosciences Influx high-speed sorter flow cytometer with a 100 μm nozzle. Unstained lung cell suspensions were used as negative gating controls for each fluorophore. Approximately 300 DAPI^–^APC^+^tdTomato^+^ live PNECs were collected into FACS buffer, and 25,000 DAPI^–^APC^+^tdTomato^–^ live non-neuroendocrine epithelial cells were also collected. Data were analyzed and plotted using FlowJo v10.

### scRNA-Seq.

Following FACS, sorted cells were counted and processed using Chromium Single Cell 3′ v3 kit (10x Genomics). cDNA quality was checked (high quality) prior to submission. Sequencing was carried out on the NovaSeq 6000 (Illumina) platform at the Institute for Genomic Medicine, UCSD. Cell Ranger package v3.0.2 (43) (10x Genomics) was used to align the raw reads onto the mouse reference genome (GRCm38) and generate the feature-barcode matrix.

R package Seurat v4.1.3 was used to perform data quality control, normalization, principal components analysis, and UMAP. Cells with fewer than 200 or more than 2,500 unique feature counts, or more than 7% mitochondrial counts, were considered “low-quality” cells and thus removed from further analysis. Doublets were removed using “doubletFinder” (44, 45). Global-scaling method “LogNormalize” was used to normalize the feature expression. A total of 2,000 top variable features were identified by the function FindVariableFeatures and selected for subsequent principal components analysis. The top 20–50 significant genes were chosen to conduct cell clustering by using the UMAP algorithm with default settings. The expression level and feature of selected genes were profiled and visualized by R package ggplot2 v4.1.3. Populations of identified immune cells, endothelial cells, erythroid cells, and remnant cells that had no distinctive cluster properties were removed from analysis to focus solely on airway epithelial cells.

### Statistics.

Cell numbers were manually counted from images taken from each section. An average of 15–25 sections from the same lung were quantified for each individual sample. As indicated in the figure legends, 1-tailed Student’s *t* test, 1-way and 2-way ANOVA, and Tukey’s multiple comparisons test were used on the data sets. Results were presented as mean ± SD. All statistical analyses were performed using Prism version 6 software (GraphPad). A *P* value of less than 0.05 was considered statistically significant. Fluorescence intensity was quantified using mean intensity density over the total area (μm^2^) on ImageJ (NIH) for Notch1 intensity levels.

### Study approval.

The animal care and experiments were performed in compliance with institutional guidelines that were reviewed and approved by the UCSD Animal Care and Use Committee and conformed to the NIH *Guide for the Care and Use of Laboratory Animals* (National Academies Press, 2011). All animals were handled according to approved Institutional Animal Care and Use Committee protocols (S16187) at UCSD.

### Data availability.

Values for all data points for each graph are included in the Supporting Data Values file. The scRNA-Seq data (raw data and annotated data) are archived in National Center for Biotechnology Information Gene Expression Omnibus (GSE273687). All data, reagents, and models are available upon request made to the corresponding author.

## Author contributions

EK and XS conceived the idea and designed the experiments. EK, BKW, YS, and HI performed all the experiments. BKW and YS conducted experiments associated with AHR and immune response in mice. HI aided in collecting data on trachea experiments. JV provided administrative, technical, and logistic support. EK, BKW, HI, and XS analyzed the data and wrote the manuscript.

## Supplementary Material

Supplemental data

Supporting data values

## Version 1 07/08/2025

Electronic publication
