# Supplementary material for: Allergen induces pulmonary neuroendocrine cell hyperplasia in a model of asthma
Source: JCI Insight. 2025 Jul 8;10(13):e187018. doi: 10.1172/jci.insight.187018 (PMC12288900; doi:10.1172/jci.insight.187018)
Supplement: Supplemental data [file jciinsight-10-187018-s209.pdf]

Allergen Induces Pulmonary Neuroendocrine Cell Hyperplasia in a Model of Asthma

Estelle Kim[1], Brian K. Wells[1], Hannah Indralingam[2], Yujuan Su[2], Jamie Verheyden[2], Xin Sun[1,2]*

Supplementary Information

Supplementary Figures S1-S19
Supplementary Legends

Kim *et al*, 2024
Supplemental Figure 1

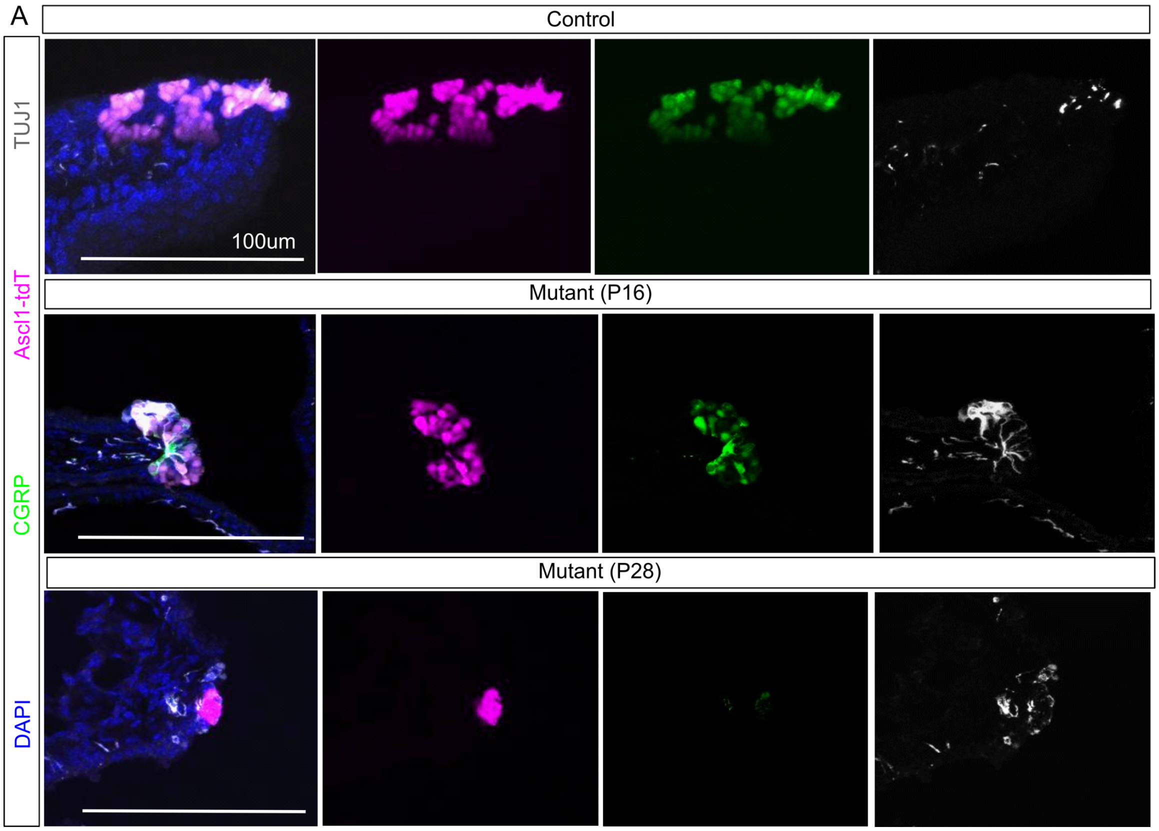

Supplemental Figure 1. PNECs are reduced in *Ascl1* mutants. A. Representative images of control (*Ascl1*$^{creERT2/+}$;*Rosa26*$^{tdTomato/+}$) and mutant (*Ascl1*$^{cre/f;}$*Rosa26*$^{tdTomato/+}$) lung sections from Figure 1B. Lungs were stained with anti-CGRP and anti-TUJ1 antibodies. Images have been separated by individual colors.

Kim et al, 2024
Supplemental Figure 2

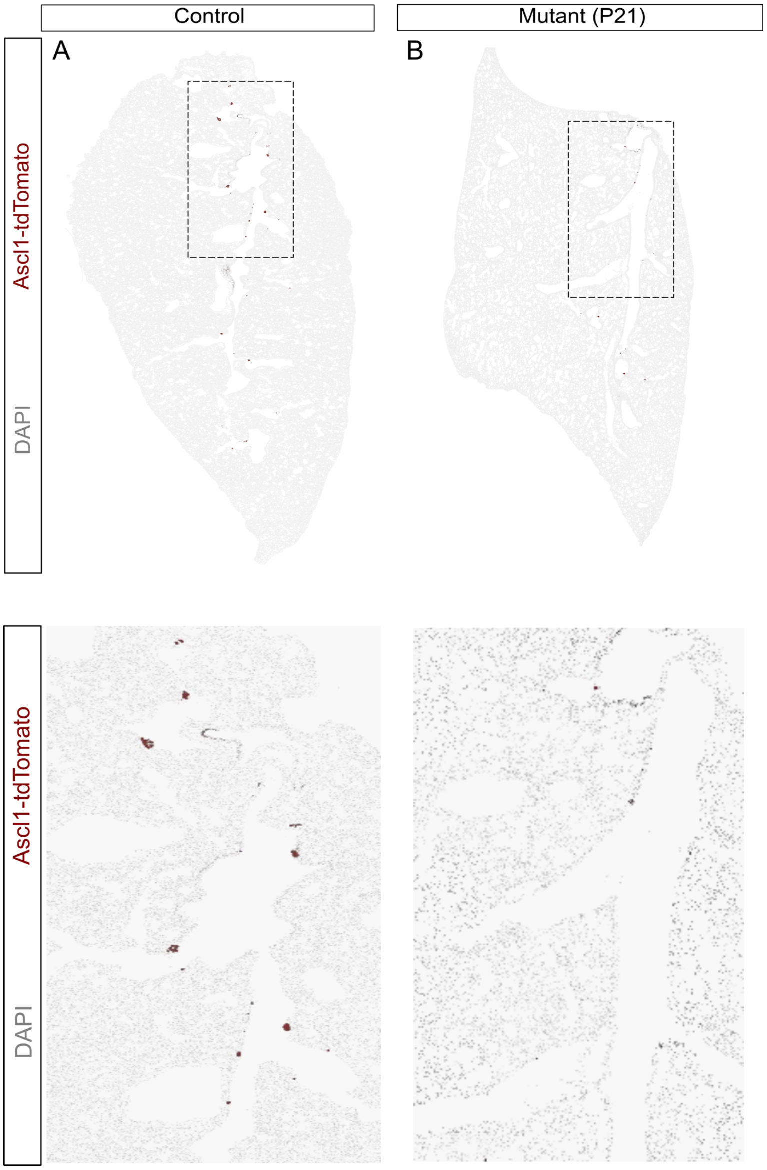

Supplemental Figure 2. *Ascl1*-lineaged cells are reduced in whole lung section. A,B. Representative images of entire control (*Ascl1*$^{creERT2/+}$;*Rosa26*$^{tdTomato/+}$) and mutant (*Ascl1*$^{cre/f;}$*Rosa26*$^{tdTomato/+}$) lung sections. Color signals as indicated.

Kim *et al*, 2024
Supplemental Figure 3

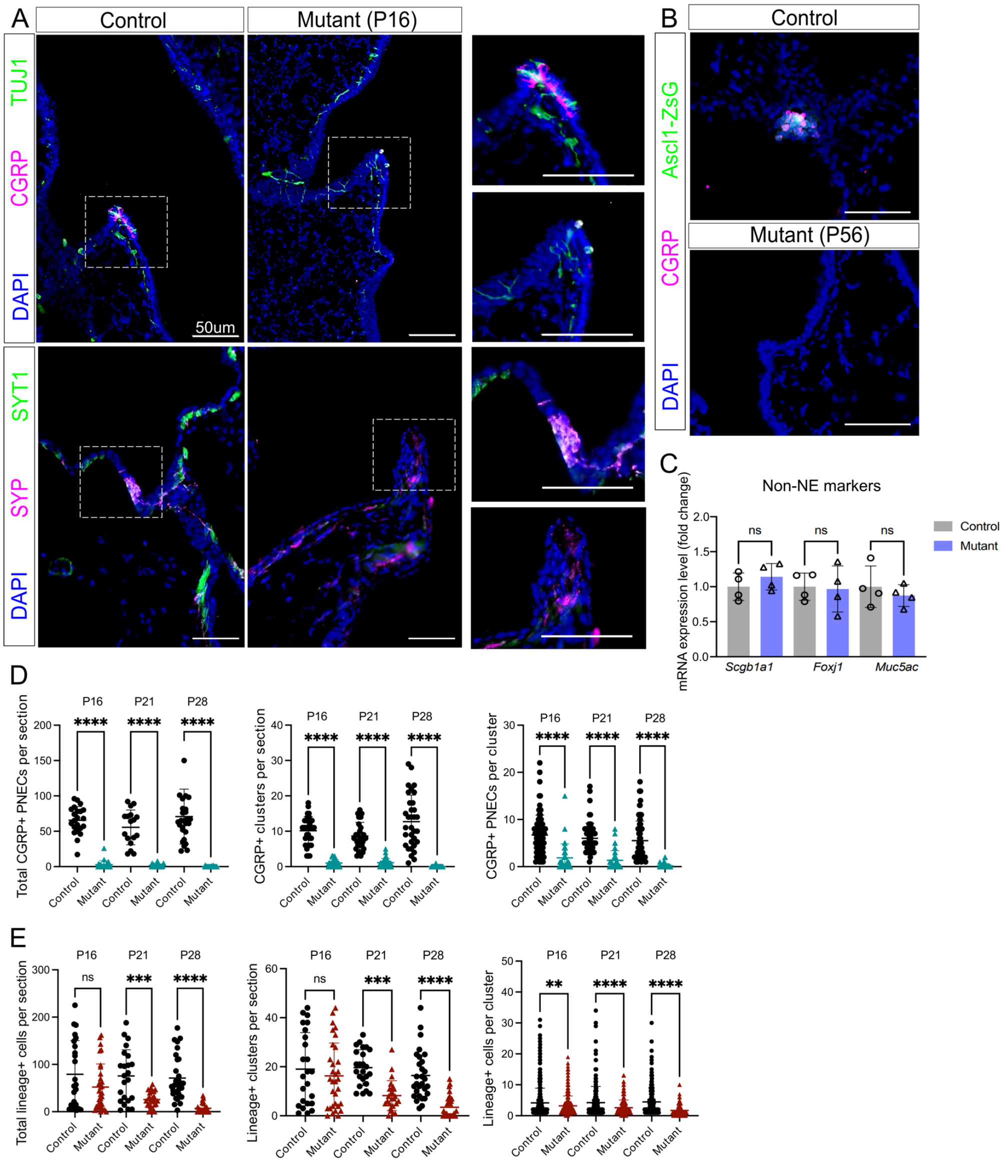

Supplemental Figure 3. PNEC number is reduced in the intrapulmonary airway of *Ascl1* mutants. A. Representative images of control (*Ascl1*$^{f/+}$) and mutant (*Ascl1*$^{cre/f}$) lung sections stained with anti-CGRP and anti-TUJ1 antibodies on top row. Representative images of control and mutant lung sections stained with anti-SYP or anti-SYT-1 antibodies on bottom row. Boxed areas are magnified. B. Representative images of control (*Ascl1*$^{creERT2/+}$;*Rosa26*$^{ZsGreen/+}$) and mutant (*Ascl1*$^{cre/f}$;*Rosa26*$^{ZsGreen/+}$) lung sections stained with anti-CGRP antibody. C. Gene expression of non-neuroendocrine airway markers as assayed by qRT-PCR in whole lungs. D,E. Quantification of PNEC number based on anti-CGRP antibody staining or *Ascl1*-lineage. Student's t-test was used (n=50-100 for each group). Each dot represents value from each section. ns for not significant ≥ 0.05, * for p<0.05, ** for p<0.01, *** for p<0.001, **** for p<0.0001. Error bars represent mean ± SD. Scale bar size as indicated.

Kim *et al*, 2024
Supplementary Figure 4

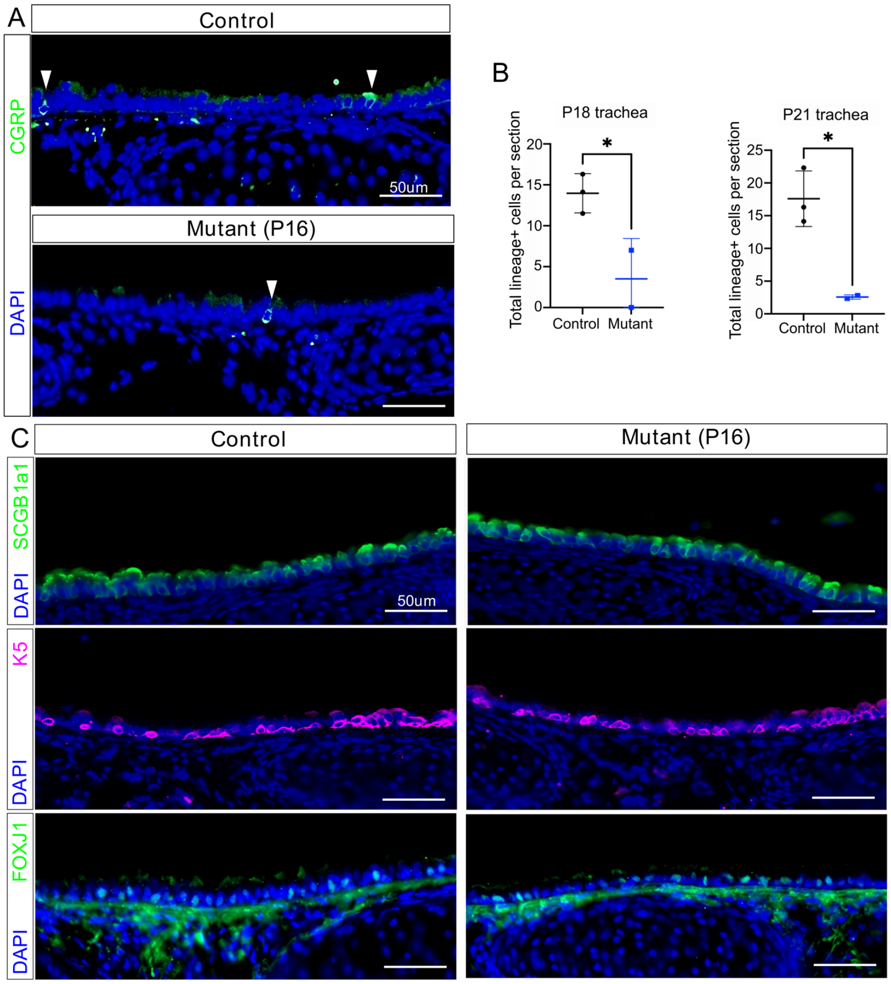

Supplemental Figure 4. PNEC number is reduced in the trachea of *Ascl1* mutants. A. Representative images of control and mutant trachea sections stained with anti-CGRP antibody. Arrowheads point to PNECs. B. Quantification of PNEC number based on *Ascl1*-lineaged cells in the trachea at indicated time points. Each dot represents the average value of multiple sections from one mouse. ns for not significant ≥ 0.05, * for p<0.05. Error bars represent mean ± SD. C. Representative images of control and mutant trachea sections stained with anti-SCGB1A1 antibody to detect club cells, anti-K5 antibody to detect basal cells, and anti-Foxj1 antibody to detect ciliated cells. Signal under the airway in Foxj1-stained lung is background. Scale bar size as indicated.

Kim *et al*, 2024
Supplemental Figure 5

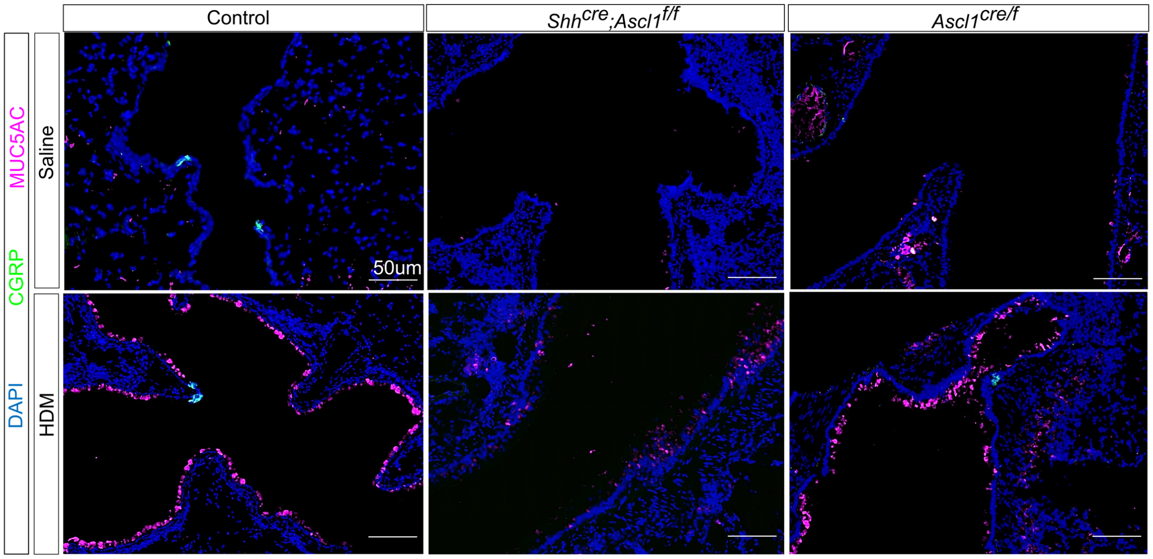

Supplemental Figure 5. *Ascl1* mutant mice display comparable allergen-induced goblet cell metaplasia compared to control. Representative images of lung sections from WT or *Ascl1* mutant mice (*Shh*^{cre};*Ascl1*$^{f/f}$ and *Ascl1*$^{cre/f}$) either subjected to saline or HDM, stained with anti-MUC5AC antibody. Scale bar size as indicated.

Kim *et al*, 2024
Supplemental Figure 6

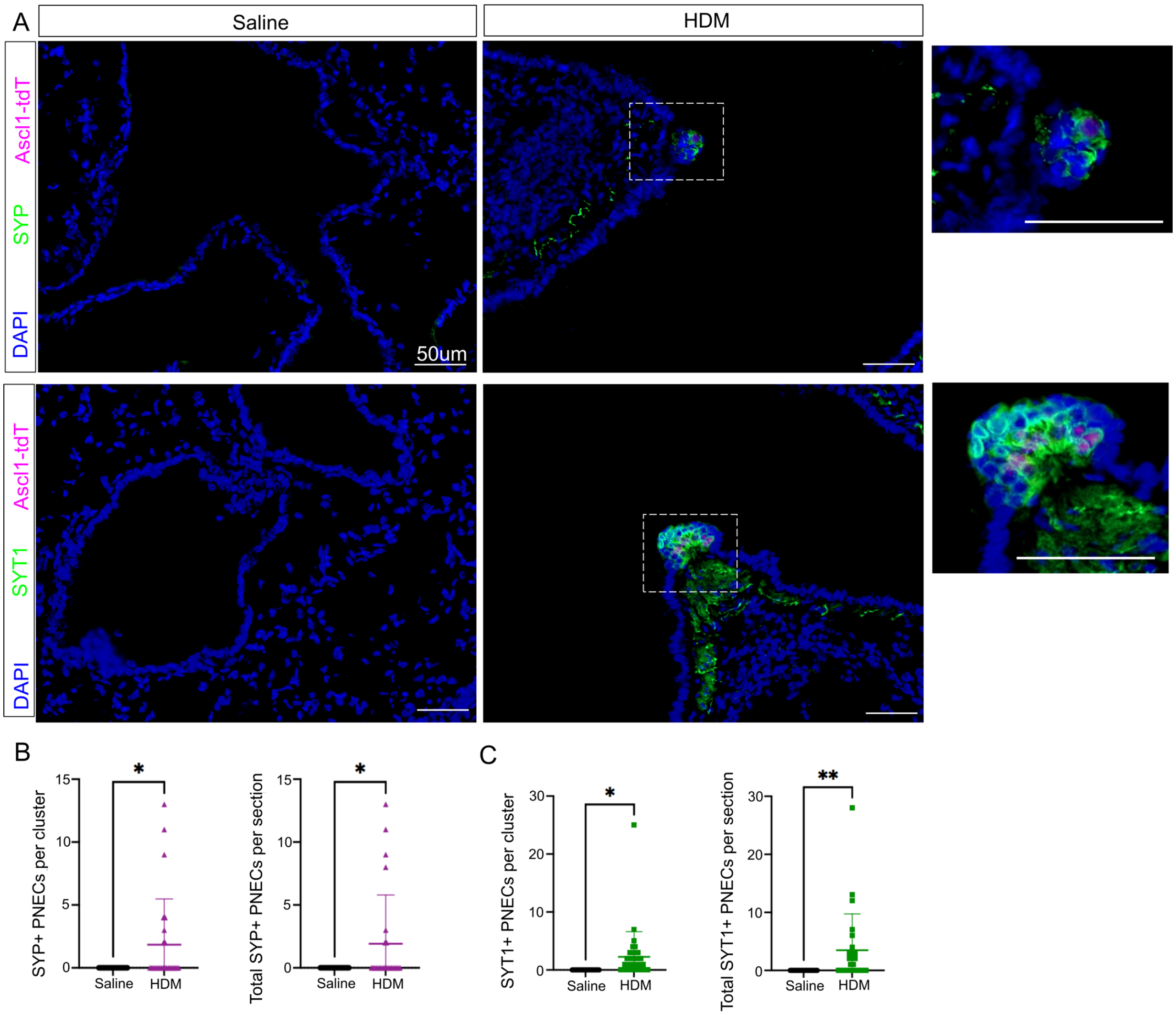

Supplemental Figure 6. Ectopic PNECs that arise in *Ascl1* mutant following HDM challenge express canonical NE markers. A. Representative images of lung sections from *Ascl1* mutant reporter (*Ascl1*$^{cre/f;}$*Rosa*$^{26tdTomato/+}$) mice stained with anti-SYP or anti-SYT-1 antibody. Boxed area is magnified. Scale bar size as indicated. B,C. Quantification of PNEC number based on either anti-SYP or anti-SYT1 antibody staining. Student's t-test was used (n=15-20 for each group). Each datapoint represents value from an individual section. * for p<0.05, ** for p<0.01. Error bars represent mean ± SD.

Supplemental Figure 7

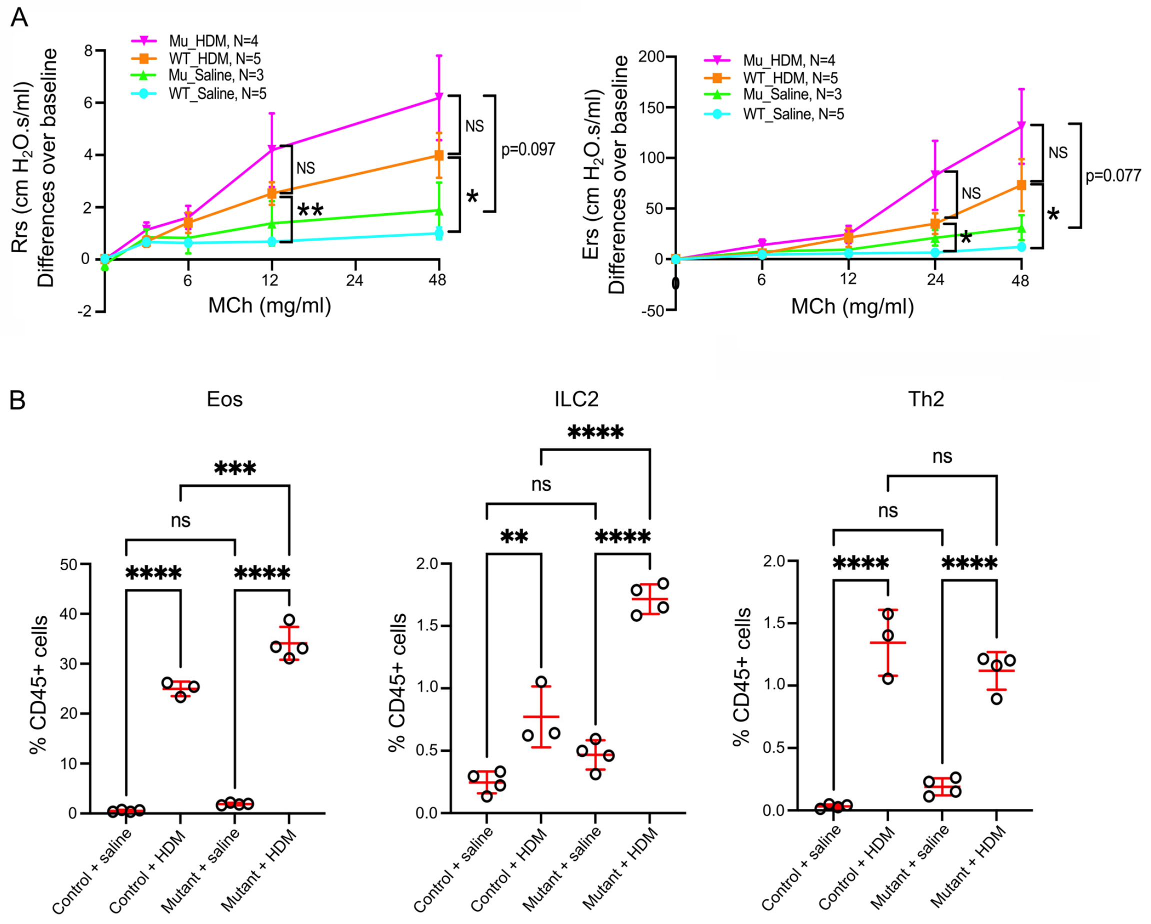

Supplemental Figure 7. AHR and immune profiling reveal heightened response to allergen challenge in *Ascl1$^{creERT2/f}$* mutants. A. Airway hyperresponsiveness (AHR) measured using the flexiVent system in control (*Ascl1$^{creERT2/+}$*) and mutant (*Ascl1$^{creERT2/f}$*) mice exposed to four doses of either saline or HDM. Each datapoint represents the value from one mouse (n=3-4 mice per condition). B. Flow cytometric quantification of eosinophils (Eos; CD45^{+}Siglec-F^{+}Ly6g^{-}CD11b^{+}), group 2 innate lymphoid cells (ILC2; Lin^{-}CD45^{+}CD127^{+}), and T helper cells (Th2; Lin-CD45+CD4+TCRb+) from lung single-cell suspensions of saline- or HDM-exposed control and mutant mice. Each datapoint represents one mouse. One-way ANOVA was used (n=3–5 per group). ns for p≥0.05, * for p<0.05, ** for p<0.01, *** for p<0.001, **** for p<0.0001. Error bars represent mean ± SD.

Kim *et al*, 2024
Supplemental Figure 8

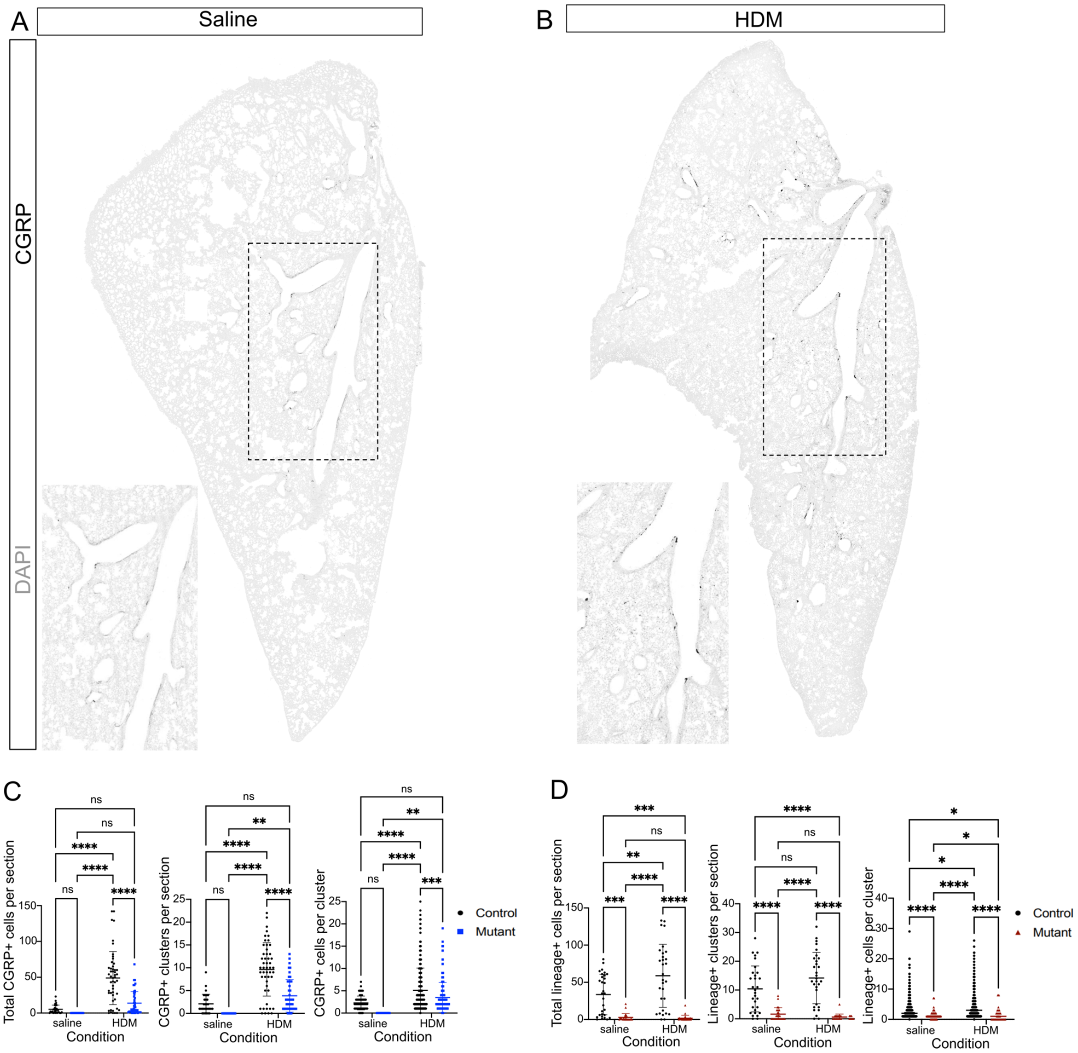

Supplemental Figure 8. Increase in PNEC number following allergen challenge. A,B. Representative images of entire WT lung sections subjected to either saline or HDM stained with anti-CGRP antibody. DAPI blue color was pseudo-colored to gray. C,D. Quantification of PNEC number based on CGRP-antibody staining or *Ascl1*-lineage, comparing control (WT) and *Ascl1* mutant (*Ascl1*$^{cre/f}$) mice subjected to either saline or HDM. Two-way ANOVA was used (n=50-100 for each group). Each datapoint represents value from each section. ns for not significant ≥ 0.05, * for p<0.05, ** for p<0.01, *** for p<0.001, **** for p<0.0001. Error bars represent mean ± SD.

Kim *et al*, 2024
Supplemental Figure 9

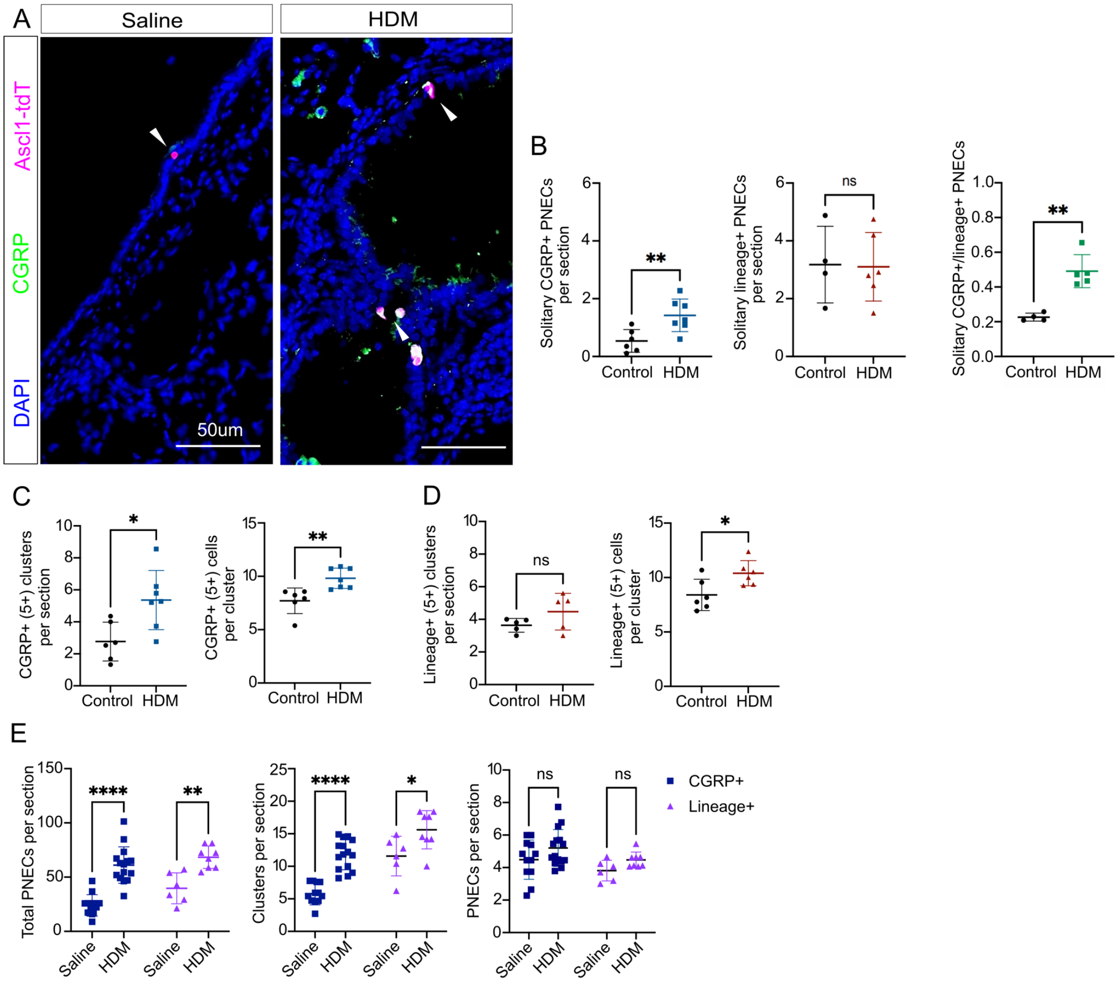

Supplemental Figure 9. Allergen challenge led to increased PNEC number as solitary cells and in clusters. A. Representative images of lung sections from *Ascl1* reporter (*Ascl1*$^{creERT2/+}$*;Rosa26*$^{tdTomato/+}$) mice exposed to saline or HDM stained with anti-CGRP antibody. B. Quantification of solitary CGRP+ PNECs, *Ascl1*-lineaged cells, and the ratio of CGRP+ PNECs to *Ascl1*-lineaged cells. C,D. Quantification of large clusters (5+ PNECs in a cluster) based on CGRP staining or *Ascl1*-lineage. Student's t-test was used (n=6-7 for each group). E. Quantification of PNEC number based on CGRP staining to *Ascl1*-lineaged cells from all doses of HDM. One-way ANOVA was used (n=6-15 for each group). ns for not significant ≥ 0.05, * for p<0.05, ** for p<0.01, **** for p<0.0001. Error bars represent mean ± SD. Scale bar size as indicated.

Kim *et al*, 2024
Supplemental Figure 10

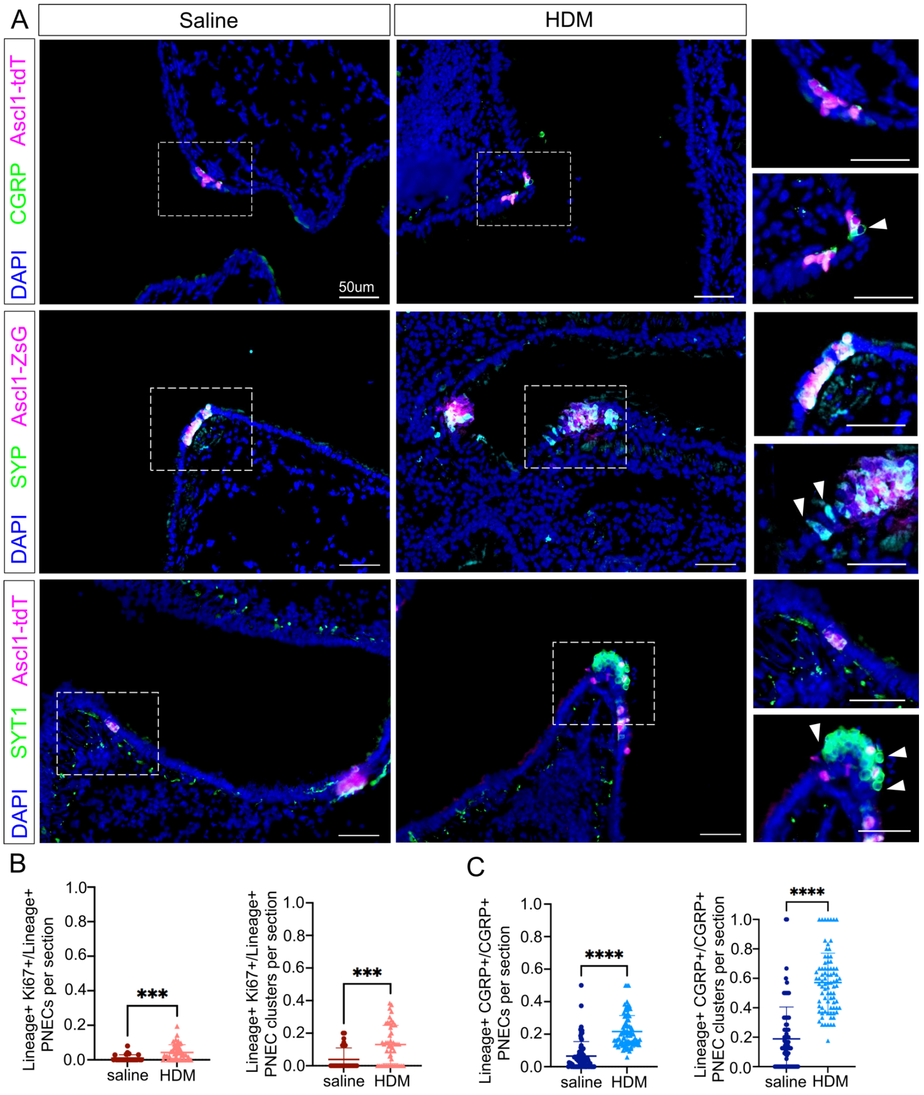

Supplemental Figure 10. Non-*Ascl1*-lineaged PNECs arise in WT mice following allergen challenge. A. Representative images of lung sections from *Ascl1* reporter (*Ascl1*$^{creERT2/+}$;*Rosa26*$^{tdTomato/+}$) mice subjected to saline or HDM, stained with either anti-CGRP, anti-SYP antibody, or anti-SYT1 antibody. Arrowheads point to non-lineaged PNECs. Boxed areas are magnified. B,C. Quantification of the overlap of either Ki67+ *Ascl1*-lineaged cells over the total of *Ascl1*-lineaged cells or SCGB1a1-lineaged CGRP+ cells over the total of CGRP+ PNECs. Student's t-test was used (n=50-100 for each group). Each datapoint represents value from an individual section. ns for not significant ≥ 0.05, * for p<0.05, ** for p<0.01, *** for p<0.001, **** for p<0.0001. Error bars represent mean ± SD. Scale bar size as indicated.

Kim *et al*, 2024
Supplemental Figure 11

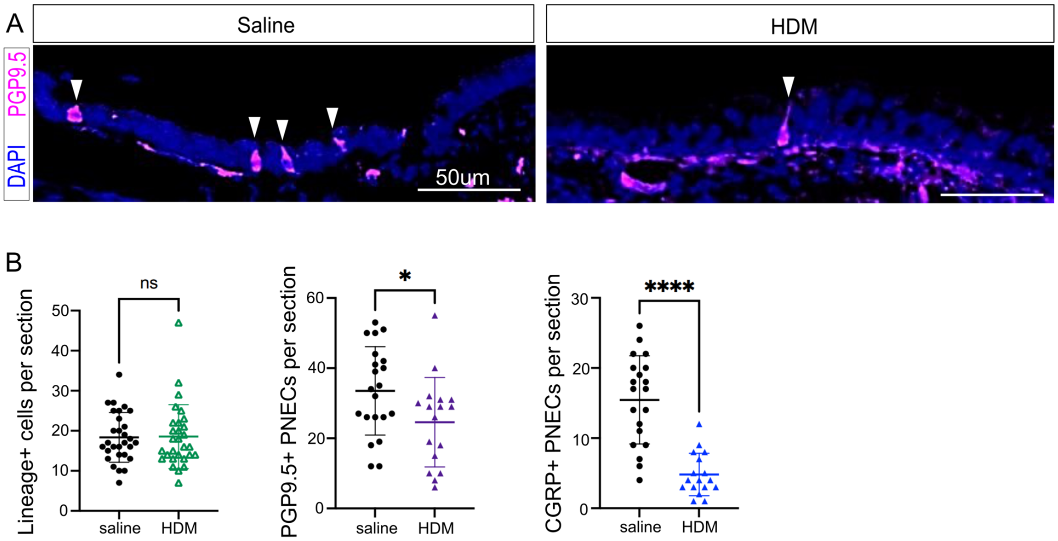

Supplemental Figure 11. Allergen challenge did not lead to PNEC hyperplasia in the trachea. A. Representative images of trachea sections exposed to saline or HDM stained with anti-PGP9.5 antibody. Arrowheads indicate PGP9.5+ PNECs. B. Quantification of PNEC numbers based on *Ascl1*-lineage or PGP9.5 staining. Student's t-test was used (n=25-40 for each group). Each datapoint represents value from each section. ns for not significant ≥ 0.05, * for $p<0.05$, **** for $p<0.0001$. Error bars represent mean ± SD. Scale bar size as indicated.

Kim *et al*, 2024
Supplemental Figure 12

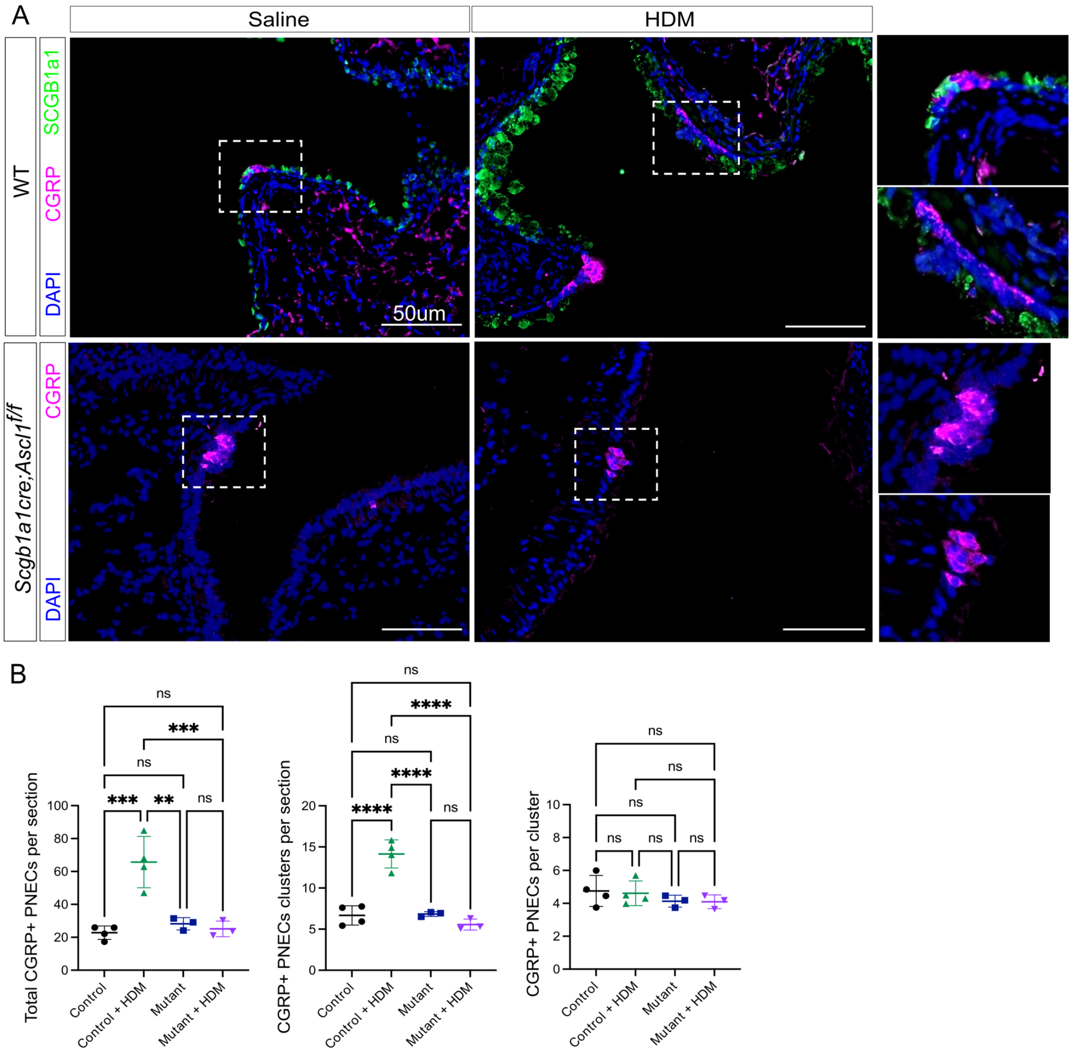

Supplemental Figure 12. *Ascl1* is required for club cell transdifferentiation in allergen response. A. Representative images of lung sections from WT mice subjected to either saline or HDM, stained with anti-CGRP and anti-SCGB1a1 antibodies on top row. There is no overlap of staining. Magenta signal under the airway is background staining. Representative images of lung sections from *Scgb1a1cre;Ascl1* mutant (*Scgb1a1*$^{creERT2/+}$;*Ascl1*$^{f/f}$) mice subjected to either saline or HDM, stained with anti-CGRP antibody on bottom row. Boxed areas are magnified. B. Quantification of PNEC numbers based on CGRP staining in control or *Scgb1a1cre;Ascl1* mutant. Each datapoint represents the average value of multiple sections from one mouse. One-way ANOVA was used (n=3-4 for each group). ns for not significant ≥ 0.05, ** for p<0.01, *** for p<0.001, **** for p<0.0001. Error bars represent mean ± SD. Scale bar size as indicated.

Kim *et al*, 2024
Supplemental Figure 13

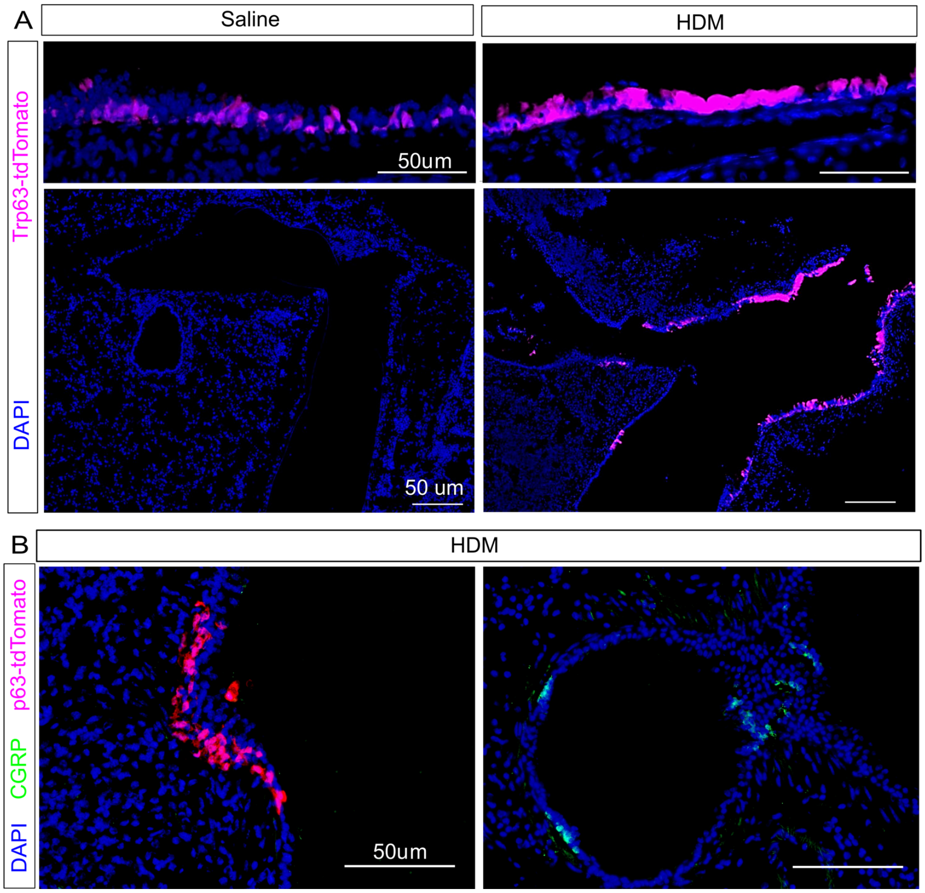

Supplemental Figure 13. Allergen-induced ectopic PNECs did not derive from basal cells. A. Representative images of trachea sections from p63 reporter (*Trp63*$^{creERT2/+}$;*Rosa26*$^{tdTomato/+}$) mice exposed to saline or HDM on top row. Representative images of lung sections from p63 reporter mouse exposed to HDM stained on bottom row. There are ectopic *Trp63*-lineaged cells in the intrapulmonary airway following allergen challenge. B. Images of *Trp63* reporter lung sections near branchpoint junctions where PNECs are located. No CGRP signal is detected in *Trp63*-lineaged region. Scale bar size as indicated.

Kim *et al*, 2024
Supplemental Figure 14

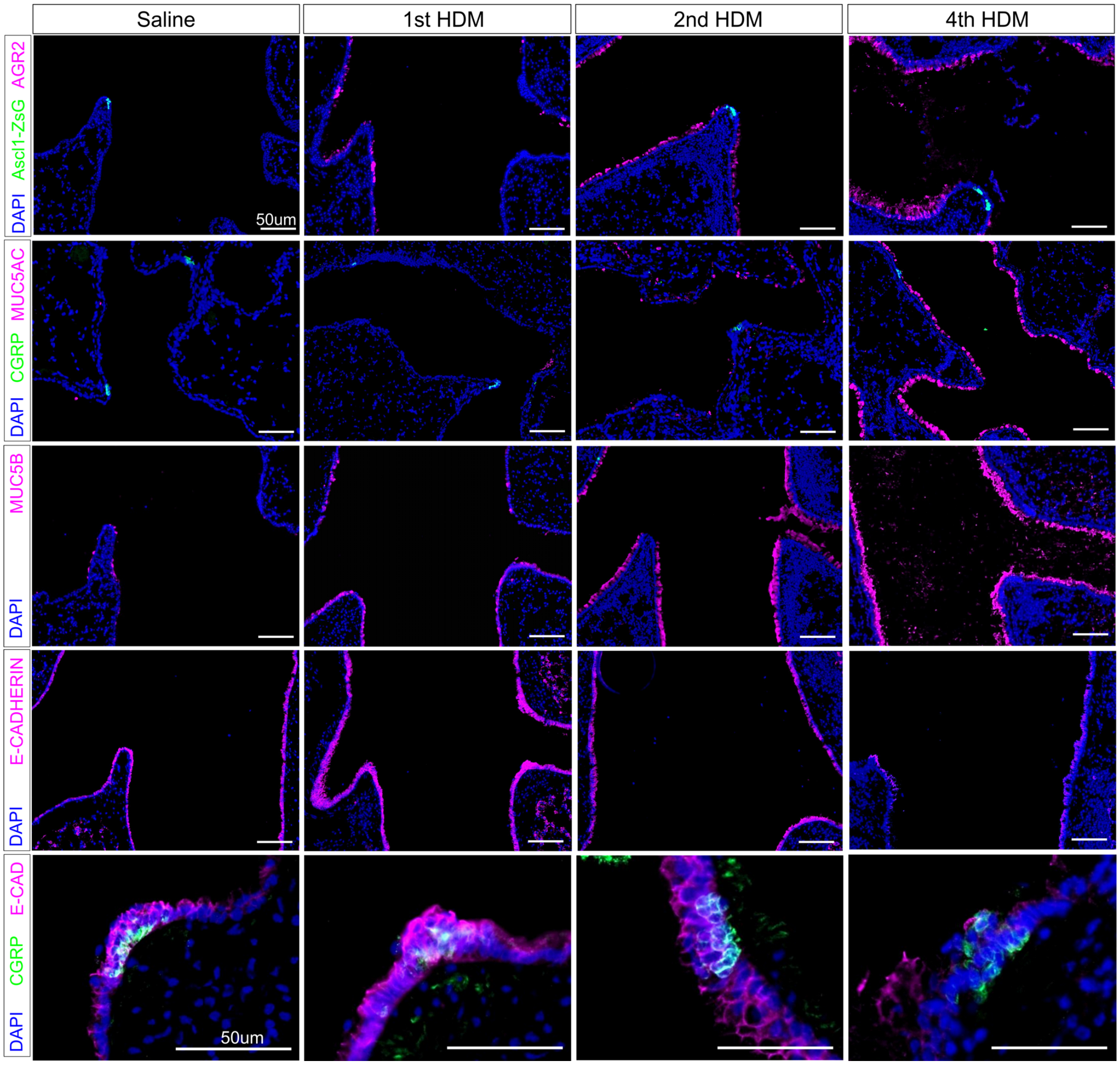

Supplemental Figure 14. Dose-dependent changes following allergen challenge. Representative images of lung sections from *Ascl1* reporter (*Ascl1*$^{creERT2/+}$;*Rosa26*$^{tdTomato/+}$) mice exposed to increasing doses of HDM stained with anti-AGR2 (top row), anti-MUC5AC (second row), and anti-MUCB (third row) antibodies. Representative images of lung sections from WT mice exposed to increasing doses of HDM stained with anti-ECAD antibody (last two rows) and anti-CGRP antibody (last row). Scale bar size as indicated.

Kim *et al*, 2024
Supplemental Figure 15

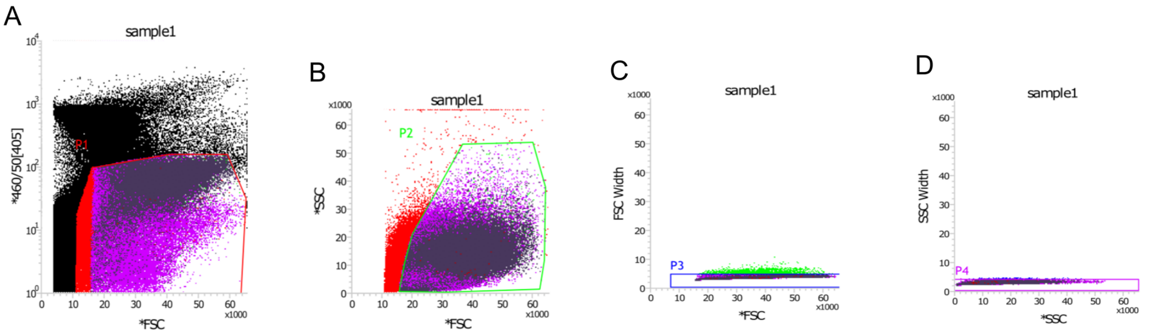

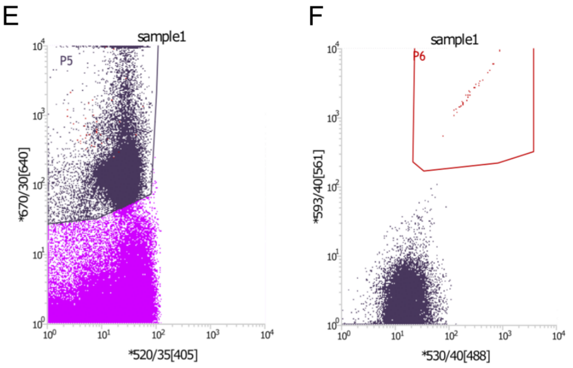

G

Populations: sample1

Populations	Even...	%Total	%Parent
All Events	200,000	100.00%	####
P1	124,143	62.07%	62.07%
P2	106,993	53.50%	86.19%
P3	105,341	52.67%	98.46%
P4	105,195	52.60%	99.86%
P5	34,014	17.01%	32.33%
P6	35	0.02%	0.10%

Supplemental Figure 15. Gating strategy to enrich epithelial cells and PNECs. A. Gating of live cells determined by DAPI staining. B. Removal of debris based on forward and side scatter gating. C,D. Sort for single cells based on forward scatter (C) and side scatter (D). E. Gating for epithelial cells based on Epcam+ signal. F. Gating for *Ascl1*-lineaged cells based on tdTomato+ signal. G. Summary of cells acquired from sequential gating strategy from a total of 200,000 events.

Kim *et al*, 2024
Supplemental Figure 16

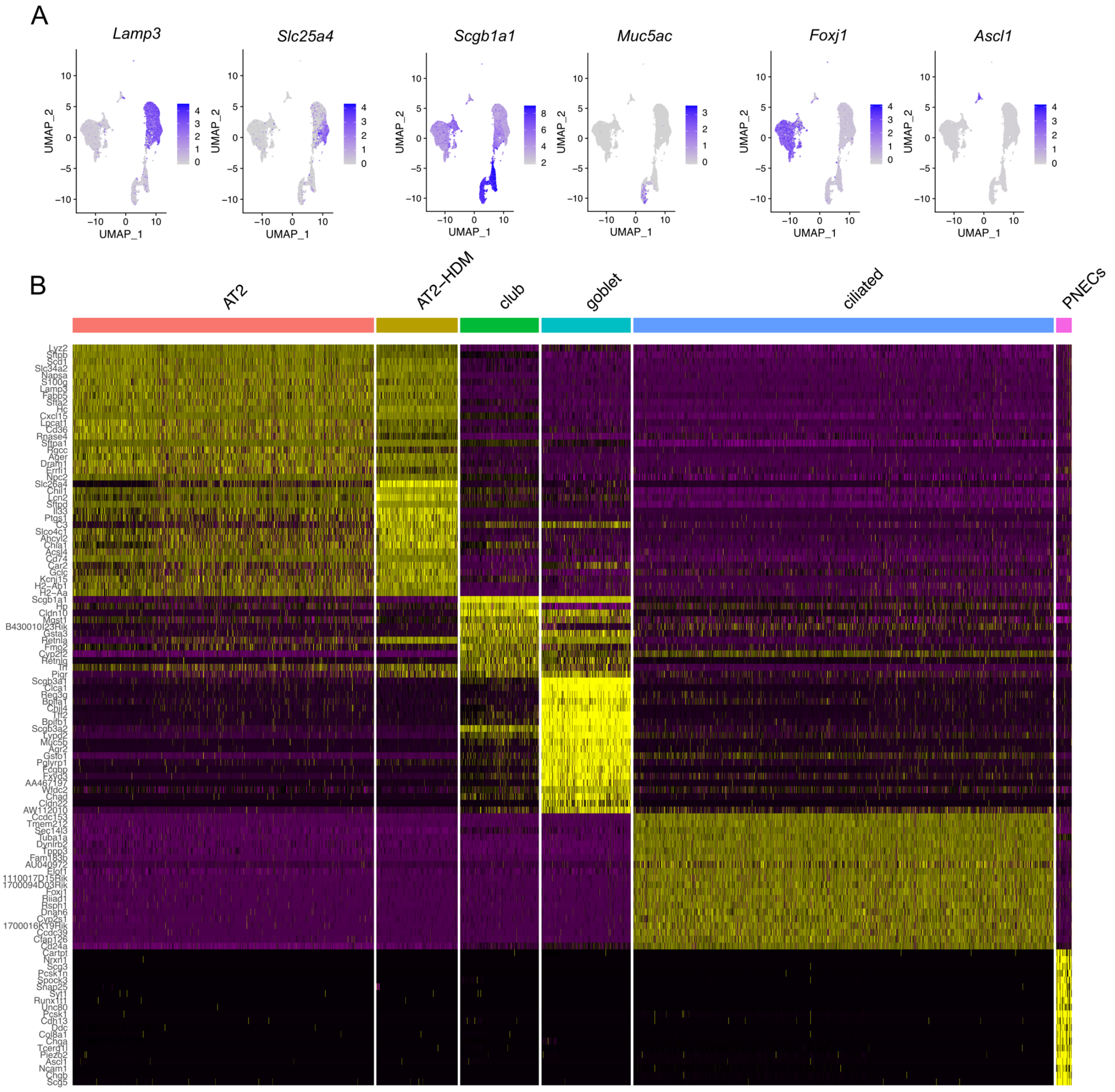

Supplemental Figure 16. Single-cell RNA-sequencing analysis of epithelial cells in saline and HDM-challenged lungs. A. Feature plots of epithelial cell type markers. B. Heatmap of top genes across all epithelial cell populations. Nine major cell clusters are labeled with the color bar at the top of the heatmap. AT2, alveolar type 2 cells; AT2-HDM, HDM-specific alveolar type 2 cells; club; goblet; ciliated; PNECs, pulmonary neuroendocrine cells.

Kim *et al*, 2024
Supplemental Figure 17

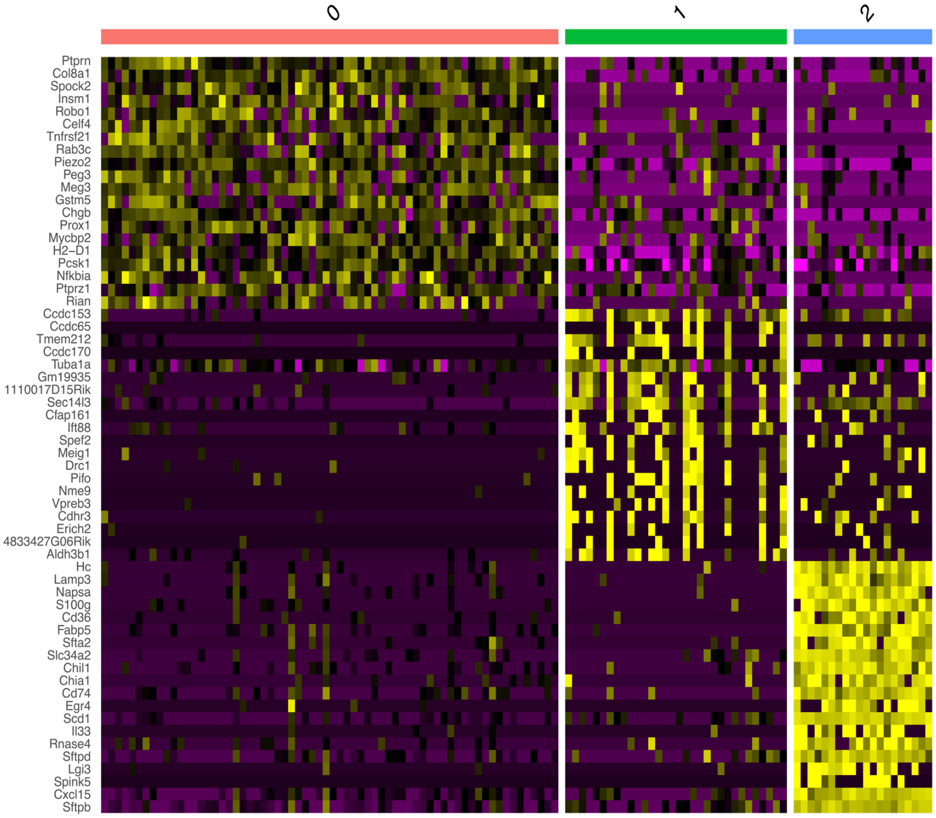

Supplemental Figure 17. Single cell RNA-sequencing analysis of PNECs in saline and HDM-challenged lungs. Heatmap of top 20 genes in PNEC population, separated by clusters.

Kim *et al*, 2024
Supplemental Figure 18

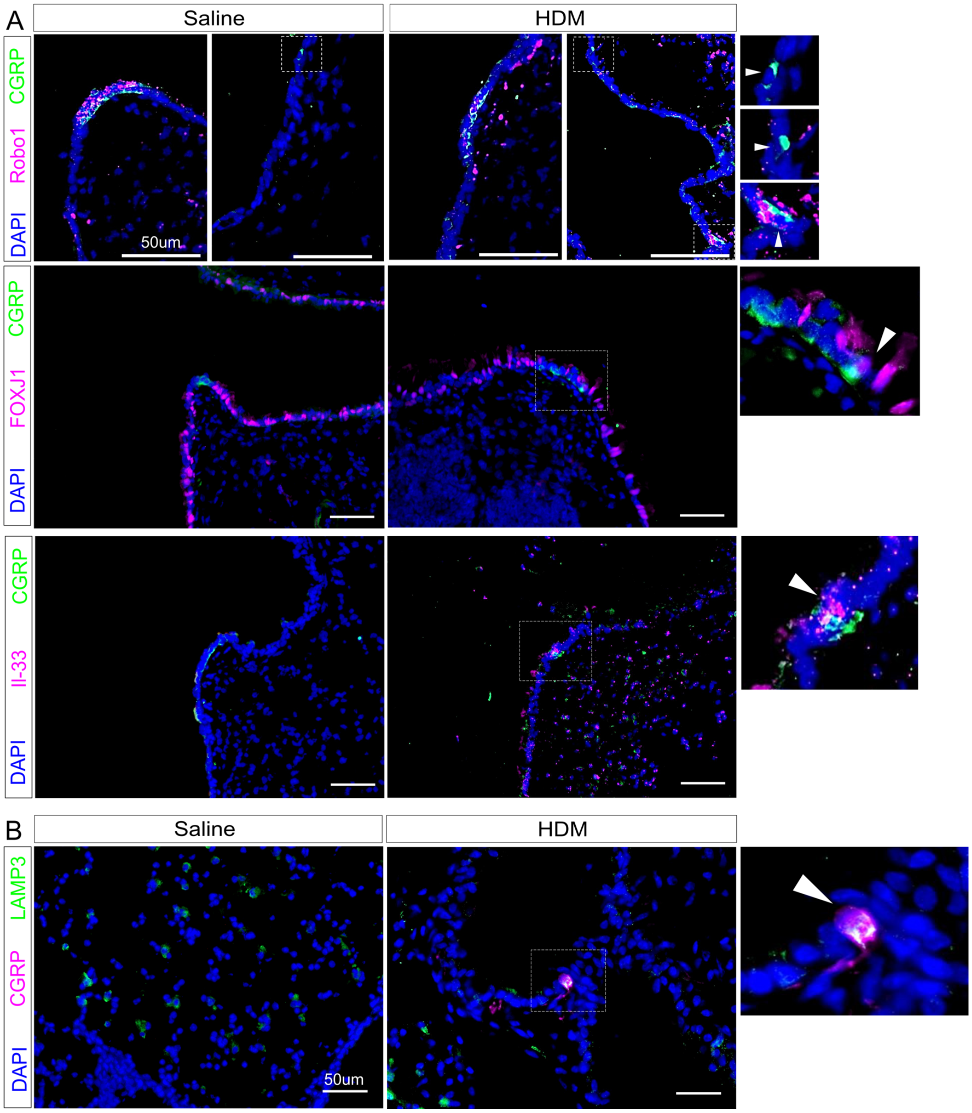

Supplemental Figure 18. Validation of PNEC heterogeneity. A. Representative images of lung sections stained with *Robo1* probes (first row), anti-FOXJ1 antibody (second row), or *Il-33* probes (last row), and anti-CGRP antibody to detect PNECs. Arrowheads point to CGRP+ cells devoid of *Robo1* expression (first row). Remaining arrowheads point to overlap of expression with CGRP+ cells. Boxed areas are magnified. Scale bar size as indicated. B. Representative images of lung sections stained with anti-LAMP3 and anti-CGRP antibodies. In saline control, LAMP3 signal is only in AT2 cells. Following HDM challenge, arrowhead points to solitary CGRP+LAMP3+ cell.

Kim *et al*, 2024
Supplemental Figure 19

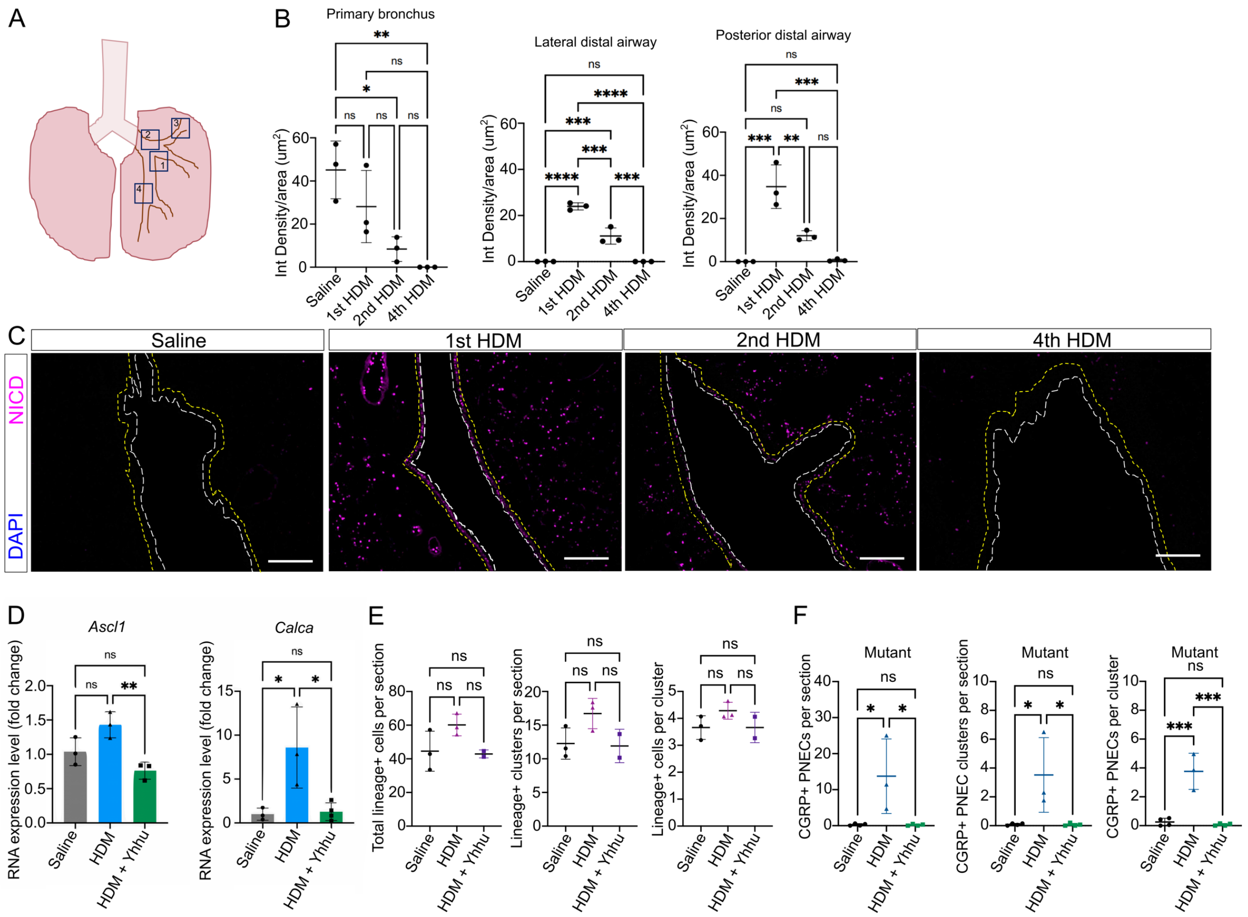

Supplemental Figure 19. Downregulation of Notch signaling contributed to PNEC increase following allergen challenge. A. Schematic of boxed regions (1-4) corresponding to different regions of the airway that were quantified, with primary branchpoint junction (1), primary bronchus (2), lateral distal airway (3), and posterior distal airway (4). B. Quantification of signal intensity over traced airway epithelial area in different regions of the airway (regions 2-4, with quantification for region 1 shown in Figure 5B). C. Representative images of lung sections in the lateral distal airway of lungs exposed to saline or different doses of HDM stained with anti-NICD antibody. D. Gene expression of *Ascl1* and *Calca* as assayed by qRT-PCR in whole adult lungs. Student's t-test was used (n=3 for each group). E. *Ascl1* reporter (*Ascl1*$^{creERT2/+}$;*Rosa26*$^{tdTomato/+}$) mice were exposed to saline, HDM, and HDM with *Notch1* agonist, Yhhu-3792. Quantification of PNEC numbers based on *Ascl1*-lineage. F. *Ascl1* mutant (*Ascl1*$^{cre/f}$) mice were exposed to saline, HDM, and HDM with *Notch1* mutant. Each datapoint represents the average value of multiple sections from one mouse. One-way ANOVA was used (n=3-4 for each group). ns for not significant ≥ 0.05, ** for p<0.01, *** for p<0.001, **** for p<0.0001. Error bars represent mean ± SD. Scale bar size as indicated.
